# Inborn Errors of Immunity in Algerian Children and Adults: A Single-Center Experience Over a Period of 13 Years (2008–2021)

**DOI:** 10.3389/fimmu.2022.900091

**Published:** 2022-04-21

**Authors:** Brahim Belaid, Lydia Lamara Mahammed, Ouardia Drali, Aida Mohand Oussaid, Nabila Souad Touri, Souhila Melzi, Abdelhak Dehimi, Lylia Meriem Berkani, Fatma Merah, Zineb Larab, Ines Allam, Ouarda Khemici, Sonya Yasmine Kirane, Mounia Boutaba, Reda Belbouab, Hadjira Bekkakcha, Assia Guedouar, Abdelhakim Chelali, Brahim Baamara, Djamila Noui, Hadda Baaziz, Radia Rezak, Sidi Mohamed Azzouz, Malika Aichaoui, Assia Moktefi, Redha Mohamed Benhatchi, Meriem Oussalah, Naila Benaissa, Amel Laredj, Assia Bouchetara, Abdelkader Adria, Brahim Habireche, Noureddine Tounsi, Fella Dahmoun, Rabah Touati, Hamza Boucenna, Fadila Bouferoua, Lynda Sekfali, Nadjet Bouhafs, Rawda Aboura, Sakina Kherra, Yacine Inouri, Saadeddine Dib, Nawel Medouri, Noureddine Khelfaoui, Aicha Redjedal, Amara Zelaci, Samah Yahiaoui, Sihem Medjadj, Tahar Khelifi Touhami, Ahmed Kadi, Fouzia Amireche, Imane Frada, Shahrazed Houasnia, Karima Benarab, Chahynez Boubidi, Yacine Ferhani, Hayet Benalioua, Samia Sokhal, Nadia Benamar, Samira Aggoune, Karima Hadji, Asma Bellouti, Hakim Rahmoune, Nada Boutrid, kamelia Okka, Assia Ammour, Houssem Saadoune, Malika Amroun, Hayet Belhadj, Amina Ghanem, Hanane Abbaz, Sana Boudrioua, Besma Zebiche, Assia Ayad, Zahra Hamadache, Nassima Ouaras, Nassima Achour, Nadira Bouchair, Houda Boudiaf, Dahila Bekkat-Berkani, Hachemi Maouche, Zahir Bouzrar, Lynda Aissat, Ouardia Ibsaine, Belkacem Bioud, Leila Kedji, Djazia Dahlouk, Manoubia Bensmina, Abdelkarim Radoui, Mimouna Bessahraoui, Nadia Bensaadi, Azzeddine Mekki, Zoulikha Zeroual, Koon-Wing Chan, Daniel Leung, Amar Tebaibia, Soraya Ayoub, Dalila Mekideche, Merzak Gharnaout, Jean Laurent Casanova, Anne Puel, Yu Lung Lau, Nacira Cherif, Samir Ladj, Leila Smati, Rachida Boukari, Nafissa Benhalla, Reda Djidjik

**Affiliations:** ^1^ Department of Medical Immunology, Beni Messous University Hospital Center, University of Algiers 1, Algiers, Algeria; ^2^ Department of Pediatrics B, Hussein Dey University Hospital Center, University of Algiers 1, Algiers, Algeria; ^3^ Department of Pediatrics A, Beni Messous University Hospital Center, University of Algiers 1, Algiers, Algeria; ^4^ Department of Pediatrics, Blida University Hospital Center, University of Blida, Blida, Algeria; ^5^ Department of Pediatrics, Bab El Oued University Hospital Center, University of Algiers 1, Algiers, Algeria; ^6^ Department of Pediatrics, Setif University Hospital Center, University of Setif 1, Setif, Algeria; ^7^ Department of Medical Immunology, Beni Messous University Hospital Center, Algiers, Algeria; ^8^ Department of Pediatrics B, Beni Messous University Hospital Center, Algiers, Algeria; ^9^ Department of Pediatrics B, Beni Messous University Hospital Center, University of Algiers 1, Algiers, Algeria; ^10^ Department of Pediatrics A, Hussein Dey University Hospital Center, University of Algiers 1, Algiers, Algeria; ^11^ Department of Pediatrics, Mustapha Pacha University Hospital Center, University of Algiers 1, Algiers, Algeria; ^12^ Department of Pediatrics, Djelfa Public Hospital Institution, Djelfa, Algeria; ^13^ Department of Pediatrics, Batna University Hospital center, University of Batna, Batna, Algeria; ^14^ Department of Pediatric Gastroenterology and Nutrition, Canastel Children’s Hospital, Oran, Algeria; ^15^ Department of Pediatric Gastroenterology and Nutrition, Canastel Children’s Hospital, University of Oran, Oran, Algeria; ^16^ Department of Pediatric Pneumo-Allergology, Canastel Children’s Hospital, Oran, Algeria; ^17^ Department of Pediatric Pneumo-Allergology, Canastel Children’s Hospital, University of Oran, Oran, Algeria; ^18^ Department of Children’s Infectious Diseases, Canastel Children’s Hospital, University of Oran, Oran, Algeria; ^19^ Department of Pediatric Hematology, Canastel Children’s Hospital, Oran, Algeria; ^20^ Department of Pediatrics, El Bayadh Public Hospital Institution, EL Bayadh, Algeria; ^21^ Department of Pediatrics, Bejaia University Hospital Center, University of Bejaia, Bejaia, Algeria; ^22^ Department of Pediatrics, Central Hospital of the Army, University of Algiers 1, Algiers, Algeria; ^23^ Department of Pediatrics, Mother & Child Hospital of Tlemcen, University of Tlemcen, Tlemcen, Algeria; ^24^ Department of Pediatrics, Saida Public Hospital Institution, Saida, Algeria; ^25^ Department of Pediatrics, El Oued Public Hospital Institution, El Oued, Algeria; ^26^ Department of Pediatrics, Barika Public Hospital Institution, Batna, Algeria; ^27^ Department of Pediatrics, Ghardaia Public Hospital Institution, Ghardaia, Algeria; ^28^ Private Practitioner, Constantine, Algeria; ^29^ Department of Pneumology A, Beni Messous University Hospital Center, University of Algiers 1, Algiers, Algeria; ^30^ Department of Pediatrics, Mother & Child Hospital of EL Mansourah, University of Constantine 3, Constantine, Algeria; ^31^ Department of Pediatrics, Biskra Public Hospital Institution, Biskra, Algeria; ^32^ Department of Pediatrics, El Harrouche Public Hospital Institution, Skikda, Algeria; ^33^ Department of Pediatrics, Tizi Ouzou University Hospital Center, University of Tizi Ouzou, Tizi Ouzou, Algeria; ^34^ Department of Pediatrics, Tighennif Public Hospital Institution, Mascara, Algeria; ^35^ Department of Pediatrics, El-Harrach Public Hospital Institution, University of Algiers 1, Algiers, Algeria; ^36^ Department of Pediatrics, Ain Oulmene Public Hospital Institution, Setif, Algeria; ^37^ Department of Pediatrics, Ain Azel Public Hospital Institution, Setif, Algeria; ^38^ Department of Pediatrics, Mother & Child Hospital of Touggourt, Touggourt, Algeria; ^39^ Department of Pneumology, Mila Public Hospital Institution, Mila, Algeria; ^40^ Department of Pediatrics, Khenchela Public Hospital Institution, Khenchela, Algeria; ^41^ Department of Pediatrics, El Khroub Public Hospital Institution, Constantine, Algeria; ^42^ Department of Pediatrics, Kolea Public Hospital Institution, Tipaza, Algeria; ^43^ Department of Infectious Diseases, EL Kettar Specialized Hospital, University of Algiers 1, Algiers, Algeria; ^44^ Department of Pediatrics, Annaba University Hospital Center, University of Annaba, Annaba, Algeria; ^45^ Department of Pediatric Oncology, Mustapha pacha University Hospital Center, University of Algiers 1, Algiers, Algeria; ^46^ Department of Pediatrics, Bologhine Public Hospital Institution, University of Algiers 1, Algiers, Algeria; ^47^ Department of Pediatrics, Mother & Child Hospital of Tipaza, University of Blida, Algiers, Algeria; ^48^ Department of Pediatrics, Ain Taya Public Hospital Institution, University of Algiers 1, Algiers, Algeria; ^49^ Department of Pediatrics B, Douera University Hospital Center, University of Blida, Algiers, Algeria; ^50^ Department of Pediatrics and Adolescent Medicine, School of Clinical Medicine, Li Ka Shing Faculty of Medicine, The University of Hong Kong, Hong Kong, Hong Kong SAR, China; ^51^ Department of Internal Medicine, El Biar Public Hospital Institution, University of Algiers 1, Algiers, Algeria; ^52^ Department of Internal Medicine, Beni Messous University Hospital Center, University of Algiers 1, Algiers, Algeria; ^53^ Department of Pneumology B, Beni Messous University Hospital Center, University of Algiers 1, Algiers, Algeria; ^54^ Laboratory of Human Genetics of Infectious Diseases, Necker Hospital for Sick Children, INSERM UMR 1163, Paris, France; ^55^ Imagine Institute, University of Paris, Paris, France; ^56^ St Giles Laboratory of Human Genetics of Infectious Diseases, Rockefeller University, New York, NY, United States; ^57^ Howard Hughes Medical Institute, New York, NY, United States; ^58^ Department of Pediatrics, El Biar Public Hospital Institution, University of Algiers 1, Algiers, Algeria

**Keywords:** primary immunodeficiency, inborn errors of immunity, Algeria, epidemiology, molecular diagnosis, clinical features, diagnosis

## Abstract

**Background:**

Inborn errors of immunity (IEI) predispose patients to various infectious and non-infectious complications. Thanks to the development and expanding use of flow cytometry and increased awareness, the diagnostic rate of IEI has markedly increased in Algeria the last decade.

**Aim:**

This study aimed to describe a large cohort of Algerian patients with probable IEI and to determine their clinical characteristics and outcomes.

**Methods:**

We collected and analyzed retrospectively the demographic data, clinical manifestations, immunologic, genetic data, and outcome of Algerian IEI patients - diagnosed in the department of medical immunology of Beni Messous university hospital center, Algiers, from 2008 to 2021.

**Results:**

Eight hundred and seven patients with IEI (482 males and 325 females) were enrolled, 9.7% of whom were adults. Consanguinity was reported in 50.3% of the cases and a positive family history in 32.34%. The medium age at disease onset was 8 months and at diagnosis was 36 months. The median delay in diagnosis was 16 months. Combined immunodeficiencies were the most frequent (33.8%), followed by antibody deficiencies (24.5%) and well-defined syndromes with immunodeficiency (24%). Among 287 patients tested for genetic disorders, 129 patients carried pathogenic mutations; 102 having biallelic variants mostly in a homozygous state (autosomal recessive disorders). The highest mortality rate was observed in patients with combined immunodeficiency (70.1%), especially in patients with severe combined immunodeficiency (SCID), Omenn syndrome, or Major Histocompatibility Complex (MHC) class II deficiency.

**Conclusion:**

The spectrum of IEI in Algeria is similar to that seen in most countries of the Middle East and North Africa (MENA) region, notably regarding the frequency of autosomal recessive and/or combined immunodeficiencies.

## Introduction

Inborn errors of immunity (IEIs), formerly known as primary immunodeficiency disorders (PIDs), form a heterogeneous group of inherited disorders that impair the development and/or function of leukocytes or other cell types involved in immunity, and often predispose patients to recurrent, persistent, or severe infections. Some patients may also display autoimmunity, autoinflammation, allergy, or malignancy (1, 2). During the last decade, advances in understanding human genetics and immunity have led to a better recognition of several immune disorders and their underlying genetic defects.

According to the 2021 IUIS classification (3), over 450 IEI have been identified as underlying diverse clinical phenotypes. These IEI are divided into 10 main categories: (I) immunodeficiencies affecting cellular and humoral immunity, (II) combined immunodeficiencies with associated or syndromic features, (III) predominantly antibody deficiencies, (IV) diseases of immune dysregulation, (V) congenital defects of phagocyte number or function, (VI) defects in intrinsic and innate immunity, (VII) autoinflammatory diseases, (VIII) complement deficiencies, (IX) bone morrow failure, (X) phenocopies of IEI.

Inheritance of IEI can be X-linked, autosomal recessive (AR), or autosomal dominant (AD); the first symptoms can manifest at birth, early childhood, or later in life. Acquired forms of IEI resulting from somatic mutations or anti-cytokines autoantibodies are increasingly identified (4).

The prevalence and incidence of IEI vary depending on the type of disorder, age, sex, ethnicity, and geographic location. At least 1 in 10,000 people are affected by IEI worldwide (1, 2, 5, 6), This number is probably underestimated due in part to the high and precocious mortality of patients before diagnosis, and to the lack of awareness and of dedicated diagnostic tools, leading to a low rate of diagnoses, particularly in developing countries. Moreover, many conditions may result from hitherto unknown IEI, as attested by the increasing speed at which new IEI are being discovered, including IEI underlying common infectious diseases such as tuberculosis or COVID-19 (4, 7–9). However, recent advances in molecular genetic technologies, particularly next-generation sequencing, have greatly improved the identification of a growing number of specific genetic defects of the immune system as well as the number of patients (4). Indeed, recent studies showed a higher prevalence worldwide, suggesting to re-assess the previous estimates of the IEI frequency in the general population (10, 11).

In order to better estimate the prevalence of these IEI in the Middle East and North Africa (MENA) region, a global survey was recently carried out and established the MENA IEI registry (12), similarly to European (13), and North American (14) and South American (15), and Asian (16, 17) series or registries. This survey reported a high frequency of IEI predominantly due to antibody deficiencies in MENA populations (12). In sub-Saharan Africa, data are extremely rare and even unavailable for most countries (18).

Interestingly, populations in the MENA region share a particularly high rate of consanguinity which might exceed 50% in some regions (19, 20). According to the MENA report, combined immunodeficiencies were the most common IEI in North Africa (Tunisia, Algeria) and in some Middle East countries (Saudi Arabia and Kuwait), while primary antibody deficiencies were the dominant disorders in most other MENA countries (12).

Algeria has an estimated population of more than 45 million of inhabitants in 2021, with a high birth rate estimated at 21.5 births per 1000 people (21). Like all MENA countries, Algeria depicts a high level of consanguinity and subsequently of autosomal recessive disorders, similar to those reported in highly consanguineous populations (20, 22).

In the current study, we retrospectively analyzed a cohort of IEI patients to enhance our understanding of IEI and to highlight the fundamental role of immunological and molecular testing to effectively identify major forms of IEI. We also aimed to describe the clinical characteristics and outcomes of patients with IEI in Algeria.

## Methods

A single-center retrospective study was carried out at the department of medical immunology of Beni Messous University Hospital Center of Algiers, Algeria, from January 2008 to September 2021. As next generation sequencing is not available routinely in Algeria, most of our patients were diagnosed according to the diagnostic criteria set by the European Society of Immunodeficiency Disorders (ESID) (23) and classified according to the IUIS criteria (1). Immunodeficiencies secondary to other conditions (e.g., human immunodeficiency virus (HIV) infection) were excluded.

### Patient Source and Enrollment

The Algerian healthcare system is a wide network of hospitals, clinics, and dispensaries. It is mainly public, accessible and free of charge to all Algerian citizens; with a recent sharp increase in the private sector.

According to their age, patients were initially assessed by pediatricians or other adult specialists, from either outpatient clinics or inpatient wards.

Any patient with clinical signs suggestive of IEI was addressed to the department of medical immunology at Beni Messous University Hospital Center, with a request form and clinical information sheet that may suggest an IEI. Request forms were examined and entered into a dedicated database.

### Data Collection and Evaluation

The request forms contained the following information:

- referral centre’s contact details
- patient’s demographics such as full name, date of birth, gender, place of birth, age at onset of suggestive symptoms, parental consanguinity, familial history of IEI, history of suspicious sibling death, patient’s history of severe, atypical, recurrent or persistent infections, patient’s immunizations
- clinical data: particular examination findings and any specific clinical phenotype (autoimmunity, lymphoproliferation, allergy, or syndromic features)
- previous work up results
- management and treatments prior to referral
- potential suspected diagnosis and investigations requested

Regarding confidentiality and patient data privacy, the collected data were listed in an electronic database with a highly restricted access.

A further written consent was obtained for the patients or their parents (for children < 18 years) before running genetic studies.

### Laboratory Assessment

Laboratory tests are a crucial to confirm the initial clinical suspicion and guide further biological investigation of IEI.

A stepwise approach for immunological diagnostic is warranted in our department and we used the following flow chart (**Supplementary Figure S1**):

#### Initial Screening Laboratory Tests

Basic screening methods are used with standard techniques such as complete blood counts; peripheral blood smear, total serum immunoglobulins (Ig) levels (i.e. IgM, IgG, IgA, IgD and IgE), IgG subclass levels, measurement of antibody responses to vaccines (mainly in predominantly antibody deficiencies), complement assays, optic microscopy examination of scalp hair (notably in pediatric patients with bamboo hair in Netherton’s Syndrome) and immunophenotype analysis of peripheral blood cells using a flow cytometry: T-cell subsets (CD3+, CD4+, CD8+), B-cells (CD19+) and natural killer (NK) cells (CD56/CD16+).

#### In-depth Immunophenotyping

An extended phenotyping of peripheral B- and T- cell compartments was performed when indicated, including naïve, effector, and memory T cell subsets (CCR7^+/-^, CD45RA^+/-^, CD45RO^+/-^), recent thymic emigrants (RTE) CD4^+^ T cells (CD45RA^+^, CD31^+^), double negative T cells (TCRαβ^+^, CD4^-^, CD8^-^), regulatory T cells (CD4^+^, CD127^-^, CD25^+^, FOXP3^+^), class-switched (CD27^+^, IgD^-^) and non-switched memory B cell subsets (CD27^+^, IgD^+^), transitional B cells (CD24^++^, CD38^++^) and plasmablasts (CD24^-^, CD38^++^).

#### Protein Specific Assays

Flow cytometry was also used to get an accurate diagnosis of immunodeficiencies associated with defects in the expression of intracellular or cell surface proteins (**Supplementary Table S1** **)**. These tests were carried-out according to the results of previous tests or based on the index of suspicion: Human Leukocytes Antigen cells- DR (HLA-DR) expression on B cells and monocytes in case of CD4^+^ T cell lymphopenia (to rule out MHC class II deficiency); WAS protein expression in T cells in case of micro-thrombocytopenia and eczema (Wiskott Aldrich Syndrome, WAS), common γ chain expression on B cells in case of T^-^B^+^NK^-^ SCID in boys; BTK protein in monocytes in case of agammaglobulinemia associated with severe decrease of B cells in males; CD79a, CD79b, CD179α, and Mu chain in bone morrow precursor B cells in case of AR agammaglobulinemia; CD40 on B cells in case of hypogammaglobulinemia with normal or elevated IgM levels, neutropenia, and opportunistic infections (hyper-IgM syndrome, HIMS); interferon-γ receptor 1 (IFN-γR1) on monocytes in case of susceptibility to infections with environmental or low virulent mycobacterial (to rule out Mendelian Susceptibility to Mycobacterial Disease, MSMD); ZAP-70 in T cells and HLA-ABC on lymphocytes in case of severe CD8+ T cell lymphopenia (CID); CD15 and CD18 on granulocytes in case of hyperleukocytosis and skin infections (leukocyte adhesion deficiencies, LAD); SAP and XIAP proteins in lymphocytes in male (in case of Epstein Barr virus infections to rule out X-linked lymphoproliferative (XLP) syndrome XLP1/2); and perforin in NK cells when suspecting a familial hemophagocytic lymphohistiocytosis (F-HLH).

#### Advanced Immunological and Functional Assays:

More specific tests were performed **(** **Supplementary Table S1** **)**, such as *in vitro* lymphocyte proliferation to assess T cell responsiveness to mitogens and TCR-dependent signaling; protein phosphorylation analysis to unmask defects in the intracellular signaling cascade such as pSTAT1 in inborn defects of the IL-12/IFN-γ axis (for suspected MSMD) or STAT1 gain-of-function (GOF), and pSTAT-3 in hyper-IgE syndrome; induction of specific activation markers such as CD40L after *in vitro* stimulation in male patients suspected of HIMS, and IL-12RB1 on activated CD4^+^ T cells in patients suspected with MSMD; dihydrorhodamine (DHR) 123 oxidation test to assess nicotinamide adenine dinucleotide phosphate (NADPH) oxidase activity (to assess chronic granulomatous disease, CGD); NK cell degranulation and cytotoxicity assays using K562 target cells to explore F-HLH.

### Genetic Analysis

In our series, genomic deoxyribonucleic acid (DNA) was extracted from whole blood according to the previously described protocol (24).

In patients with clinical and laboratory findings indicative of a specific IEI, Sanger sequencing was performed on specific genes with high frequency of mutations and low exon numbers (*BTK*, *WAS*, *RAG1*/*2*, *ITGB1*, *CYBB*, *RFXANK*). Patients presenting clinical and biological findings suggestive of DiGeorge syndrome or Jacobsen syndrome were subjected to a high-resolution cytogenetic study using G-banding and fluorescence *in situ* hybridization (FISH) for 22q11.2 deletion or 11q23 deletion, respectively.

For patients with unusual clinical presentation, if Sanger sequencing was unable to detect any candidate rare variants, next generation sequencing (NGS) through targeted gene sequencing panels or whole exome/genome sequencing was then performed.

### Statistical Analysis

Statistical analysis was done using a commercially available software package (SPSS Statistics 17.0.0; SPSS, Chicago, Ill). Descriptive statistics were reported for all variables in the cohort. All quantitative variables were reported with mean values, and median for continuous variables. Kaplan-Meier curve plots was used to distinguish survival curves; and log-rank tests were used to compare these survival curves. A p-value of 0.05 or less (p ≤ 0.05) was considered as statistically significant.

## Results

### Frequency and Distribution of IEIs

**Figure 1:**
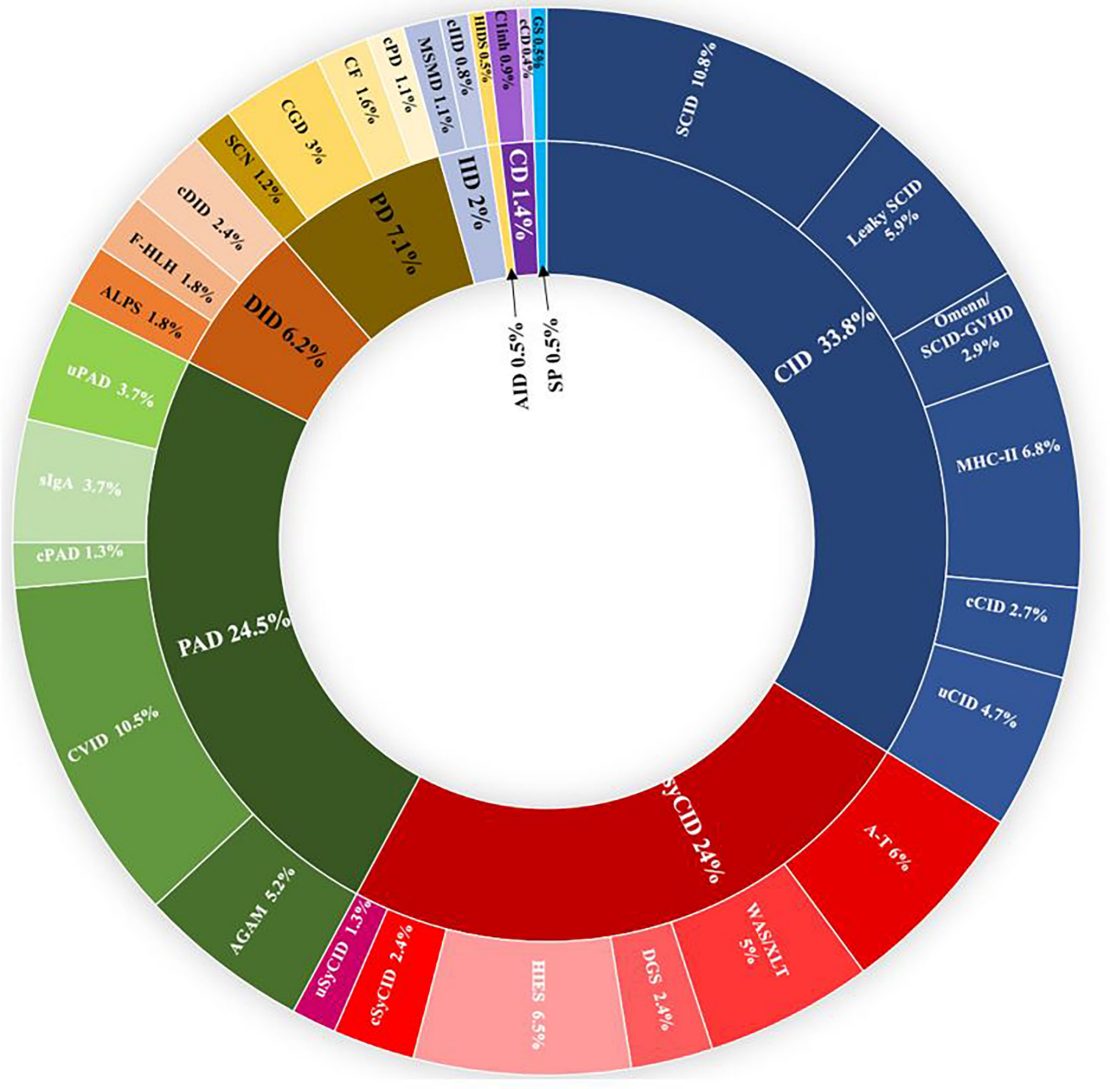
Distribution of the frequencies of IEIs according to IUIS categories in 807 Algerian patients in the study. IEI groups are shown according to IUIS classification, 2021. Total number of patients and percentage of all registered patients are shown for each group. AGAM, agammaglobulinemia; ALPS, autoimmune lymphoproliferative syndrome; AID, autoinflammatory diseases; A-T, ataxia- telangiectasia; c, classified; CD, complement deficiencies; CF, cystic fibrosis; CGD, chronic granulomatous disease; CID, combined immunodeficiencies; CVID, common variable immunodeficiency; DGS, DiGeorge syndrome, DID, Dysregulation of immune diseases; f-HLH, familial Hemophagocytic lymphohistiocytosis; GS, good syndrome; HIES, hyper-IgE syndrome; HIDS, hyper-IgD syndrome; IID, innate immune deficiencies; MSMD, mendelian susceptibility to mycobacterial disease; PAD, predominantly antibody deficiencies; PD, phagocytic deficiencies; SCN, severe congenital neutropenia; sIgA; selective IgA deficiency; SP, Somatic phenocopies; SynCID, syndromic combined immunodeficiencies; u, unclassified; WAS, Wiskott Aldrich syndrome; XLT, X linked thrombocytopenia.

From January 2008 to December 2021, more than 5,500 patients were screened for IEI in the department of medical immunology of Beni Messous University Hospital Center of Algiers. Among them, 807 were identified with confirmed IEI and included in the dedicated database. Up to 50 different IEI classified were identified, belonging to nine major categories (**Figure 1**) and, surprisingly, no patient was identified with an inherited bone marrow failure. The frequency and characteristic phenotypes of patients in each IEI category are represented in **Table 1**. The predominant category was the combined immunodeficiency (CID) (273 patients, 33.8%), followed by predominantly antibody deficiencies (PAD) (198 patients, 24.5%), combined immunodeficiencies with associated or syndromic features (SyCID) (194 patients, 24%), congenital defects of phagocytosis (57 patients, 7.1%), diseases of immune dysregulation (DID) (50 patients, 6.2%), innate immunity disorders (16 patients, 2%), complement deficiencies (11 patients,1.4%), and, lastly, autoinflammatory diseases and phenocopies of IEI in similar proportions (4 patients, 0.5%). In particular, within the CID category, SCID was the most commonly found in 10.8% (87 patients), with a predominance of T^-^B^-^NK^+^ SCID phenotype 6.8% (55 patients), followed by MHC-II deficiency (55 patients; 6.8%), hypomorphic SCID (47; 5.9%), and Omenn syndrome/SCID-GVHD (24; 2.9%). Some patients (4.7%) did not match with any specific phenotype of the CID according to the IUIS classification and were categorized as unclassified CID (uCID).

**Table 1:** General characteristics of patients with different phenotypes of inborn error of immunity.

IEI subgroup	Diagnosis	Number of cases(% total)	Sex (M/F)	Age at the onset (months),median (range)	Age at the diagnosis(months), median (range)	Diagnosis delay (months),median (range)	Parental consanguinity(%)^*^	Family history(%)^*^
**Immunodeficiencies affecting cellular and humoral immunity**		**273 (33.8%)**	**156/117**	**4 (0-360)**	**7.5 (0-456)**	**4 (0-255)**	**181(66.3%)**	**120 (43.9%)**
	▪ **SCID**	**87 (10.8%)**	**50/36**	**2 (0-7)**	**4 (0.1-15)**	**2 (0-10)**	**65 (74.7%)**	**50 (57.4%)**
	- *T^-^, B^+^, NK^+^ SCID*	*6 (0.7%)*	*5/1*	*1.08 (0-4)*	*2.75 (0.1-7)*	*1.67 (0-5.77)*	*5 (83.3%)*	*5 (83.3%)*
	- *T^-^, B^-^, NK^+^ SCID*	*55 (6.8%)*	*29/26*	*2 (0-7)*	*4.5 (0.5-9)*	*2 (0-5)*	*42 (76.3%)*	*30 (54.5%)*
	- *T^-^, B^+^, NK^-^ SCID*	*14 (1.7%)*	*10/4*	*3 (0-5)*	*4.2 (2-14)*	*2 (0.4-10)*	*7 (50%)*	*10 (71.4%)*
	- *T^-^, B^-^, NK^-^ SCID*	*12 (1.3%)*	*6/6*	*2 (0-6)*	*4 (0.5-15)*	*2.5 (0.2-9)*	*11 (91.6%)*	*5 (41.6%)*
	▪ **Hypomorphic SCID**	**47 (5.9%)**	**32/15**	**7 (0-144)**	**34 (3-240)**	**24 (0-96)**	**30 (63.8%)**	**15 (31.9%)**
	▪ **Omenn syndrome/SCID-GVHD**	**24 (2.9%)**	**10/14**	**1.4 (0-7)**	**3.75 (0-10)**	**2.5 (0-4.67)**	**17 (70.8%)**	**12 (50%)**
	▪ **MHC II deficiency**	**55 (6.8%)**	**33/22**	**5 (0-36)**	**15 (0.1-156)**	**7 (0-132)**	**34 (61.8%)**	**19 (34.5%)**
	▪ **Partial CD3 chain defects**	**3 (0.3%)**	**3/0**	**9 (8-84)**	**36 (15-96)**	**12 (6-28)**	**1 (33.3%)**	**3 (100%)**
	▪ **CD40/CD40L deficiency**	**6 (0.7%)**	**4/2**	**7 (3-24)**	**37.5 (6.5-120)**	**32 (0.5-112)**	**2 (33.3%)**	**3 (50%)**
	- *CD40 deficiency*	*2 (0.2%)*	1/1	*16 (8-84)*	*60.0 (48-72)*	*44 (40-48)*	*2 (100%)*	*2 (100%)*
	- *CD40L deficiency*	*4 (0.5%)*	4/0	*5.5 (3-12)*	*21 (6.5-120)*	*17 (0.5-112)*	*0 (0%)*	*1 (25%)*
	▪ **CD4 deficiency**	**1 (0.1%)**	**1/0**	**/**	**/**	**/**	**1 (100%)**	**0 (0%)**
	▪ **CD8 deficiency**	**1 (0.1%)**	**1/0**	**/**	**/**	**/**	**1 (100%)**	**0 (0%)**
	▪ **ZAP70 deficiency**	**2 (0.2%)**	**2/0**	**4.5 (4-5)**	**7.5 (7-8)**	**3 (2-4)**	**2 (100%)**	**1(50%)**
	▪ **DOCK8 deficiency**	**6 (0.7%)**	**3/3**	**18 (4-48)**	**66 (36-156)**	**61.5 (6-108)**	**5 (83.3%)**	**5 (83.3%)**
	▪ **IKBKB deficiency**	**3 (0.3%)**	**3/0**	**1 (0-1)**	**3 (1.5-5)**	**2 (1.5-4)**	**3 (100%)**	**3 (100%)**
	▪ **Unclassified CID**	**38 (4.7%)**	**21/17**	**9.5 (0.5-372)**	**33 (2-456)**	**24 (0-255)**	**20 (52.6%)**	**10 (26.3%)**
**Combined immunodeficiencies with associated or syndromic features**		**194 (24.0%)**	**130/64**	**9 (0-204)**	**36 (0-288)**	**24 (0-164)**	**90 (46.4%)**	**78 (40.2%)**
	▪ **Louis-Bar Syndrome**	**49 (6.0%)**	**28/21**	**25 (2-204)**	**84 (16-180)**	**38 (0-156)**	**35 (71.4%)**	**22 (44.9%)**
	▪ **WAS/XLT**	**41 (5.0%)**	**41/0**	**3 (0 -36)**	**11 (0-216)**	**5 (0-108)**	**0 (0%)**	**27 (65.8%)**
	- *WAS*	*37 (4.5%)*	*37/0*	*4 (0.5-36)*	*11 (1-216)*	*5 (0-200)*	*0 (0%)*	*24 (64.8%)*
	- *XLT*	*4 (0.4%)*	*4/0*	*2 (0-3)*	*21 (0-36)*	*18 (0-35)*	*0 (0%)*	*3 (75%)*
	▪ **DiGeorge syndrome**	**20 (2.4%)**	**14/6**	**5 (0.2-120)**	**20 (1.5-184)**	**11 (1-72)**	**5 (25%)**	**2 (10%)**
	▪ **Hyper-IgE syndrome**	**53 (6.5%)**	**38/15**	**12 (0.5-108)**	**57 (15-288)**	**36 (2-280)**	**29 (54.7%)**	**11 (30.1%)**
	- *STAT3 LOF deficiency*	*5 (0.6%)*	*4/1*	*3 (1-23)*	*72 (17-144)*	*49 (2-141)*	*2 (40%)*	*1 (20%)*
	- *CARD 11 deficiency*	*1 (0.1%)*	*1/0*	*/*	*/*	*/*	*1 (100%)*	*0 (0%)*
	- *Comel-Netherton syndrome*	6 (0.7%)	*0/6*	*2.5 (1-12)*	*5.25 (3.5-120* **)**	*3.5 (1.5-108*)	*5 (83.3%)*	*5 (83.3%)*
	▪ **EPG5 deficiency**	**7 (0.8%)**	**2/5**	**6.5 (1-12)**	**10 (1-18)**	**4 (0-12)**	**5 (71.4%)**	**2 (28.5%)**
	▪ **Chr 11q deletion syndrome**	**4 (0.4%)**	**2/2**	**24 (2-108)**	**84 (1-180)**	**42 (2-108)**	**3 (75%)**	**3 (75%)**
	▪ **Cartilage Hair Hypoplasia**	**3 (0.3%)**	**1/2**	**12 (5-24)**	**20 (17-72)**	**12 (8-48)**	**1 (33.3%)**	**2 (66.6%)**
	▪ **CHARGE Syndrome**	**1 (0.1%)**	**0/1**	**/**	**/**	**/**	**1 (100%)**	**0 (0%)**
	▪ **ORAI-1 deficiency**	**1 (0.1%)**	**1/0**	**/**	**/**	**/**	**0 (0%)**	**0 (0%)**
	▪ **Schimke immuno-osseous dysplasia**	**1 (0.1%)**	**0/1**	**/**	**/**	**/**	**0 (0%)**	**0 (0%)**
	▪ **Anhidrotic ectodermal dysplasia**	**3 (0.3%)**	**3/0**	**8 (0.5-14)**	**18 (17-36)**	**16.5 (10-22)**	**1 (33.3%)**	**2 (66.6%)**
	▪ **Undetermined syndromic CIDs**	**11 (1.3%)**	**6/5**	**4 (1.5-60)**	**31 (7.5-168)**	**22 (4-164)**	**9 (81.8%)**	**3 (27.2%)**
**Predominantly antibody deficiencies**		**198 (24.5%)**	**112/86**	**48 (3-588)**	**96 (5-660)**	**32 (0-360)**	**65 (32.8%)**	**20 (10.1%)**
	▪ **Agammaglobulinemia**	**42 (5.2%)**	**30/12**	**12 (3-120)**	**31(5-312)**	**12 (0-192)**	**20 (47.6%)**	**8 (19%)**
	- *XLA*	*18 (2.2%)*	18/0	*12.5 (4-120)*	*42 (6-312)*	*19 (0-192)*	*4 (22.2%)*	*2 (11.1%)*
	- *AR agammaglobulinemia*	*23 (2.8%)*	12/11	*9.5 (3-72)*	*22 (5-168* **)**	*10 (0-144)*	*16 (66.6%)*	*6 (25%)*
	- *AD agammaglobulinemia*	*1 (0.1%)*	*0/1*	*/*	*/*	*/*	*0 (0%)*	*0 (0%)*
	▪ **CVID**	**85 (10.5%)**	**43/42**	**180 (24-528)**	**288 (48-660)**	**60 (0-360)**	**24 (28.2%)**	**7 (8.2%)**
	- *CD19 deficiency*	*1 (0.1%)*	0/1	**/**	**/**	**/**	*1(100%)*	*0 (0%)*
	- *BAFF-R deficiency*	*1 (0.1%)*	0/1	**/**	**/**	**/**	*0 (0%)*	*0 (0%)*
	- *NFKB2 deficiency*	*1 (0.1%)*	0/1	**/**	**/**	**/**	*0 (0%)*	*0 (0%)*
	▪ **Hyper-IgM syndrome**	**9 (1.1%)**	**7/2**	**9 (4-360)**	**36 (7-408)**	**25 (0-62)**	**6 (66.6%)**	**0 (0%)**
	▪ **IgG subclass deficiency**	**2 (0.2%)**	**0/2**	**66.0 (48-84)**	**237 (162-312)**	**171 (78-264)**	**1 (50%)**	**0 (0%)**
	▪ **Selective IgA deficiency**	**30 (3.7%)**	**15/15**	**48 (12-300)**	**108 (84-444)**	**48 (0-156)**	**10 (33.3%)**	**4 (13.3%)**
	▪ **Unclassified Antibody Deficiency**	**30 (3.7%)**	**15/15**	**16.5 (3-148)**	**36 (9-156)**	**20 (0-60)**	**0 (0%)**	**0 (0%)**
**Diseases of immune dysregulation**		**50 (6.2%)**	**28/22**	**6 (1-204)**	**35.75 (0.5-492)**	**9.5 (0-342)**	**37 (74%)**	**19 (38%)**
	▪ **ALPS**	**15 (1.8%)**	**9/6**	**36 (3-120)**	**48 (10-180)**	**7 (0-108)**	**9 (60%)**	**1 (6.6%)**
	▪ **FHL**	**15 (1.8%)**	**8/7**	**5 (0.5-72)**	**6 (0.5-72)**	**1 (0-29.5)**	**12 (80%)**	**9 (60%)**
	▪ **Chediak-Higashi syndrome**	**6 (0.8%)**	**2/4**	**5 (2.5-28)**	**51 (17-84)**	**43 (13-81.5)**	**4 (66.6%)**	**2 (33.3%)**
	▪ **Griscelli syndrome**	**7 (0.8%)**	**5/2**	**6 (1.5-30)**	**20 (7-168)**	**13.5 (0-162)**	**5 (71.4%)**	**3 (42.8%)**
	▪ **APECED**	**3 (0.3%)**	**1/2**	**48 (6-204)**	**348 (96-492)**	**288 (48-342)**	**3 (100%)**	**0 (0%)**
	▪ **RIPK1 deficiency**	**4 (0.4%)**	**3/1**	**1 (1-2)**	**60 (36-126)**	**58.5 (35-125)**	**4 (100%)**	**3 (75%)**
**Congenital defects of phagocyte number or function**		**57 (7.0%)**	**34/23**	**5 (0.5-202)**	**24 (2-372)**	**8 (0-170)**	**26 (45.6%)**	**15 (26.3%)**
	▪ **Severe Congenital Neutropenia**	**10 (1.2%)**	**6/4**	**2 (1-6)**	**9.5 (2-26)**	**6 (0-23)**	**4 (40%)**	**0 (0%)**
	▪ **Chronic granulomatous disease**	**25 (3.0%)**	**18/7**	**24 (1.5-202)**	**52 (2-372)**	**12 (0-170)**	**9 (36%)**	**6 (24%)**
	- X-CGD	8 (0.9%)	8/0	*13 (1.5-80)*	*18 (3-144)*	*7.5 (1-64)*	*0 (0%)*	*2 (25%)*
	- AR-CGD	17 (2.1%)	10/7	*24 (1.5-202)*	*60 (2-372)*	*18 (0-170)*	*9 (52.9%)*	*4 (23.5%)*
	▪ **Leukocyte adhesion deficiency**	**7 (0.8%)**	**4/3**	**1 (0.5-2)**	**3 (2-54)**	**2.4 (1-53.5)**	**6 (85.7%)**	**3 (42.8%)**
	- LAD-1	6 (0.8%)	*3/3*	*1 (0.5-2* **)**	*4 (2-54)*	*2.7 (1-53.5)*	*5 (83.3%)*	*3 (50%)*
	- LAD-3	1 (0.1%)	*1/0*	*/*	*/*	*/*	*1 (100%)*	*0 (0%)*
	▪ **Cystic fibrosis**	**13 (1.6%)**	**5/8**	**2 (1-6)**	**12 (4-120)**	**8 (2-117)**	**6 (46.1%)**	**6 (46.1%)**
	▪ **GATA2 deficiency**	**2 (0.2%)**	**1/1**	**54.5 (7-102)**	**162 (60-264)**	**107.5 (53-162)**	**1 (50%)**	**0 (0%)**
**Defects in intrinsic and innate immunity**		**16 (1.9%)**	**13/3**	**6 (1-60)**	**31.5 (2-252)**	**23.5 (1-204)**	**9 (56.2%)**	**4 (25%)**
	▪ **MSMD**	**9 (1.1%)**	**7/2**	**7 (3-48)**	**30 (4-252)**	**23 (1-204)**	**7 (77.7%)**	**2 (22.2%)**
	- IL12Rβ1 deficiency	*6 (0.8%)*	*5/1*	*7 (1-48)*	*19 (18-42)*	*17 (6-37)*	*5 (83.3%)*	*1 (16.6%)*
	- STAT1 (AD-LOF) deficiency	*1 (0.1%)*	*1/0*	*/*	*/*	*/*	*1 (100%)*	*1 (100%)*
	▪ **CMC**	**5 (0.6%)**	**4/1**	**6 (2-60)**	**84 (19-156)**	**34 (13-150)**	**2 (40%)**	**2 (40%)**
	- STAT1 (AD-GOF) deficiency	3 (0.3%)	*2/1*	*6 (2-12)*	*36 (19-84)*	*34 (13-72)*	*2 (66.6%)*	*0 (0%)*
	▪ **Isolated congenital asplenia**	**1 (0.1%)**	**1/0**	**/**	**/**	**/**	**0 (0%)**	**0 (0%)**
	▪ **MECP2 deficiency syndrome**	**1 (0.1%)**	**1/0**	**/**	**/**	**/**	**0 (0%)**	**0 (0%)**
**Autoinflammatory diseases**	▪ **Hyper IgD syndrome**	**4 (0.5%)**	**1/3**	**19 (9-72)**	**79 (48-96)**	**50.5 (12-70)**	**1 (25%)**	**0 (0%)**
**Complement deficiencies**		**11 (1.3%)**	**4/7**	**120 (5-240)**	**264 (5-408)**	**120 (0-285)**	**2 (18.1%)**	**5 (45.4%)**
	▪ **C1 inhibitor deficiency**	**8 (0.9%)**	**3/5**	**102 (12-204)**	**252 (36-384)**	**132 (19-285)**	**0 (0%)**	**4 (50%)**
	▪ **C3 deficiency**	**1 (0.1%)**	**0/1**	**/**	**/**	**/**	**1 (100%)**	**0 (0%)**
	▪ **Atypical HUS**	**2 (0.2%)**	**1/1**	**102.5 (5-200)**	**134.5 (5-264)**	**32 (0-64)**	**1 (50%)**	**1 (50%)**
**Phenocopies of IEI**	▪ **Good syndrome**	**4 (0.5%)**	**4/0**	**516 (240-720)**	**522 (276-720)**	**6 (0-36)**	**0 (0%)**	**0 (0%)**

*: %: (patients/Number of patients in each category) X 100, F, Female; M, male.

AD, autosomal dominant; ALPS; autoimmune lymphoproliferative syndrome; APECED, Autoimmune polyendocrinopathy with candidiasis and ectodermal dystrophy; AR, autosomal recessive; CGD, chronic granulomatous disease; CHARGE, Coloboma, Heart defect, Atresia choanae, Retarded growth and development, Genital hypoplasia, Ear anomalies/deafness; CID, combined immunodeficiency, CMC, chronic mucocutaneous candidiasis; CVID, Common variable immune deficiency; FHL, Familial hemophagocytic lymphohistiocytosis; HUS, hemolytic uremic syndrome; IL, Interleukin; LAD, Leukocyte Adhesion deficiency; MecP2, mutations of the methyl-CpG-binding protein 2; MSMD, Mendelian susceptibility to mycobacterial disease; RIPK1, receptor interacting serine/threonine kinase-1; SCID, Severe combined immunodeficiency; SCN, severe congenital neutropenia; STAT, Signal Transducer and Activator of Transcription; WAS, Wiskott-Aldrich Syndrome; XLA, X linked agammaglobulinemia; XLT, X-linked thrombocytopenia.

Among patients with antibody deficiencies, common variable immunodeficiency (CVID) was the most common diagnosis in 10.5% (85 patients), followed by congenital agammaglobulinemia in 5.2% (42 patients). Interestingly, AR agammaglobulinemia was more common than X-linked recessive forms (2.8%, 23 patients versus 2.2%, 18 patients, respectively).

Syndromic combined immunodeficiencies were mainly represented by hyper-IgE syndrome (HIES, 53 cases,6.5%), followed by ataxia-telangiectasia (49 patients; 6.0%) and Wiskott-Aldrich syndrome (37 boys; 4.5%).

Finally, CGD, autoimmune lymphoproliferative syndrome (ALPS), f-HLH, MSMD, hereditary angioedema type I (due to C1-inhibitor deficiency), were the main other IEI.

### Patient Characteristics

There were 482 male patients (59.7%) and 325 female patients (40.3%), and all of them were Algerian. The male-to-female ratio (M/F) was close to 1.5:1. The majority of IEI categories were observed in children. However, adult patients only belonged to certain IEI groups such as predominantly antibody deficiencies, diseases of immune dysregulation, phenocopies and complement deficiencies.

**Figure 2:**
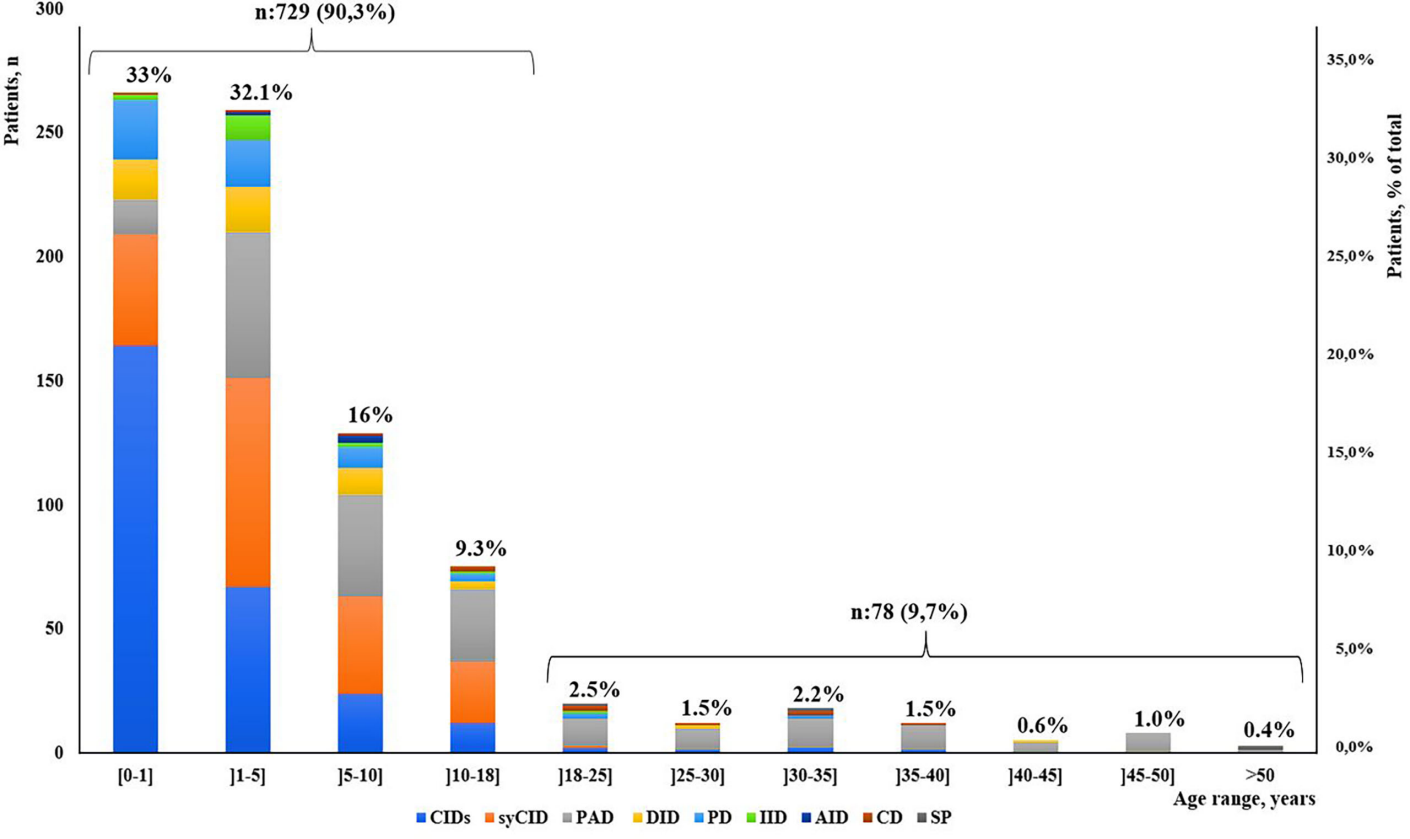
Distribution of patients according to IEI diagnosis age groups and main IEI categories (N = 807).

**Figure 3:**
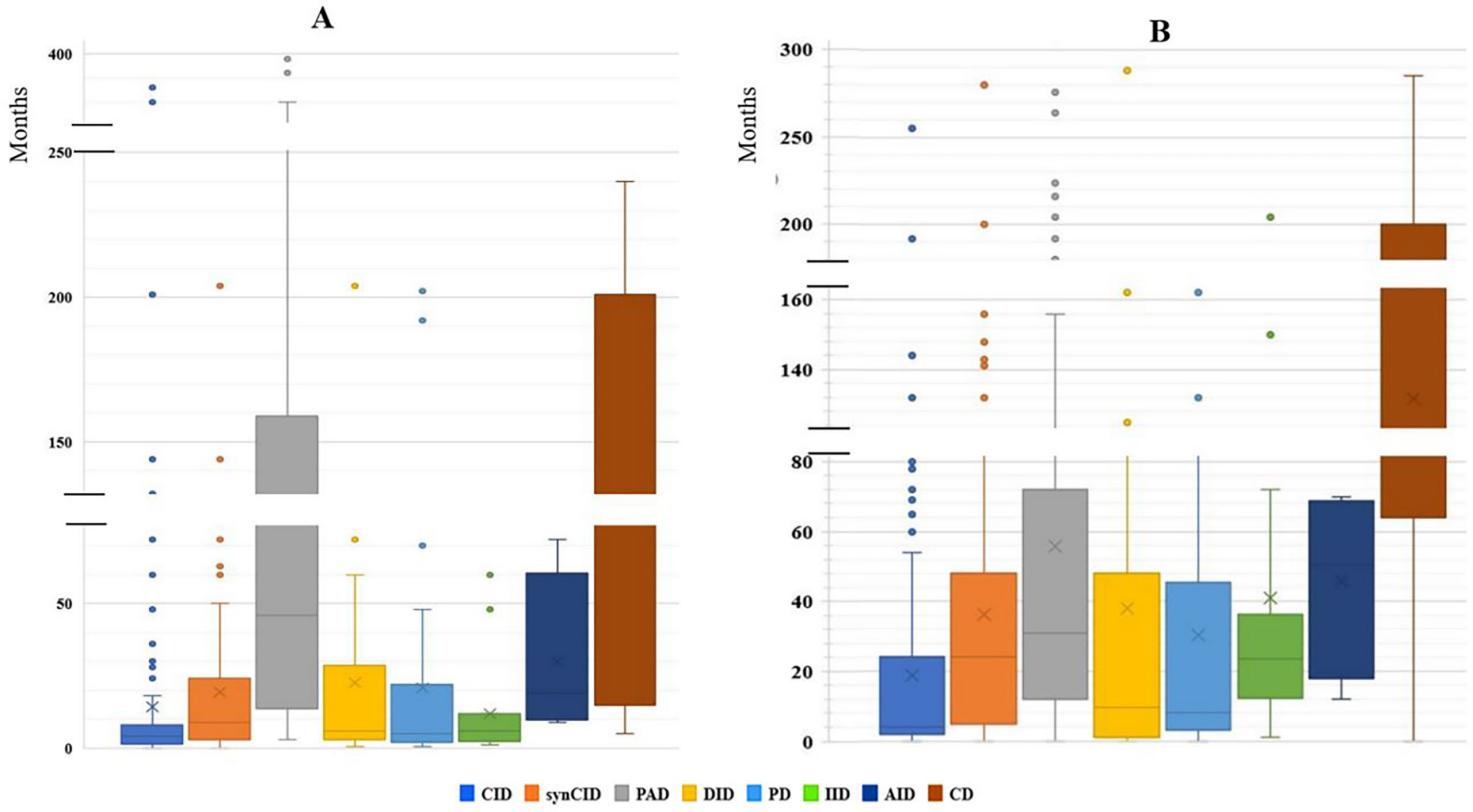
The median age of disease onset and diagnostic delay in the main IEI categories. **(A)** Median age of disease onset. **(B)** Median of diagnostic delay.

Our study indicated that age at onset, age at diagnosis, and diagnosis delay varied significantly for different types of IEI. In all patients, the median age at diagnosis was 36 months (range, 0 days to 60 years). Of these patients, 729 (90.3%) were children (mean age at diagnosis, 46.2 months), and 78 (9.7%) were adults (mean age at diagnosis, 32.9 years). The majority of the children (525 of 729, 72%) were under 5 years old (**Figure 2**). Overall, the onset of symptoms occurred at the median age of 8 months (range, 0 month to 52 years). In children, the age at onset of symptoms was less than 6 months for 316 of them (43.3%), from 6 months to 3 years for 279 (38.2%) and more than 3 years for 134 (18.3%). The symptoms (**Figure 3A**) were notably observed earlier in CID, phagocytic defects, diseases of immune dysregulation, and defects in intrinsic and innate immunity with the median onset age of 4, 5, 6 and 6 months, respectively, compared to other categories of IEI. Age at diagnosis was shorter in CID with the median diagnosis time of 7.5 months. Moreover, patients with PAD displayed symptoms remarkably later with the median age at the onset of 48 months among children, whereas in adults, this delay was observed in patients with complement deficiencies and phenocopies with the median age at the onset of 10 years and 43 years respectively (**Table 1**).

The diagnostic delay is defined as the time interval between the onset of symptoms and confirmed diagnosis of a disease. Significant diagnostic delay has been noted in Algeria (**Figure 3B**), with an overall median diagnostic delay of 16 months but over a broad age-range (from less than 1 month to 30 years), and it was more important in adult patients (9 years; 0-30 years) than children (12 months; 0-200 months). There were no antenatal diagnoses among patients registered in the database. The delay in diagnosis was shorter in CID (median: 4 months; range: 0-456 months) when compared to other groups of IEI. Among the most common CID, the shortest mean diagnostic delay was observed in SCID (median: 2 months; range: 0–10 months), followed by the Omenn syndrome/SCID-GVHD (median: 2.5 months, range: 0–4.67 months), MHC class II deficiency (median: 7 months; range: 0-132 months), and hypomorphic SCID (median: 24 months; range: 0-96 months).

Four hundred ten patients (50.3%) were born to consanguineous marriages, in most cases from first cousin parents. The rate of consanguinity varied substantially among the groups of IEI. The highest reported rates of consanguinity have been found in patients with diseases of immune dysregulation at 74%, followed by CIDs at 66.3%, while in the other categories the rates were as follow: defects in intrinsic and innate immunity (56.2%), syndromic CID (46.4%), phagocytic defects (45.6%), and predominantly antibodies deficiencies (32.8%). The lowest rates of consanguinity were recorded for phenocopies, complement deficiencies and autoinflammatory diseases at 0%, 18.1% and 25%, respectively. A positive family history of unexplained death or family member known to have an immunodeficiency disorder was noticed in 261 patients (32.34%), with the highest frequencies in patients with complement deficiencies, CID, and syndromic CID in 5 (45.4%), 120 (43.9%), and 78 (40.2%), respectively.

### Epidemiology and Geographical Distribution of IEI Patients

**Figure 4:**
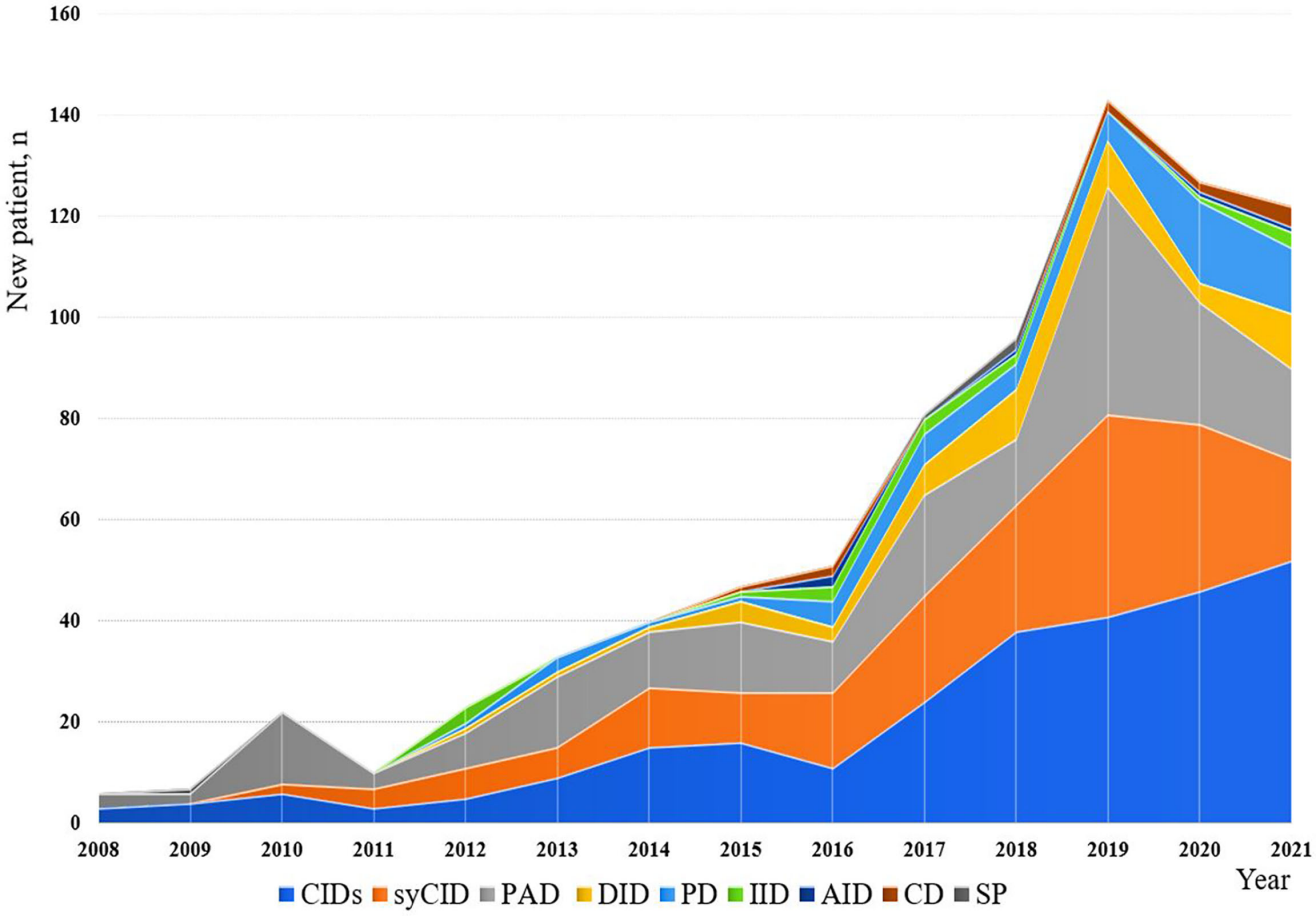
Annual numbers of newly diagnosed IEI cases from 2008 to September 2021. The registration of patients into the database has been stopped in September for the year 2021.

**Figure 5:**
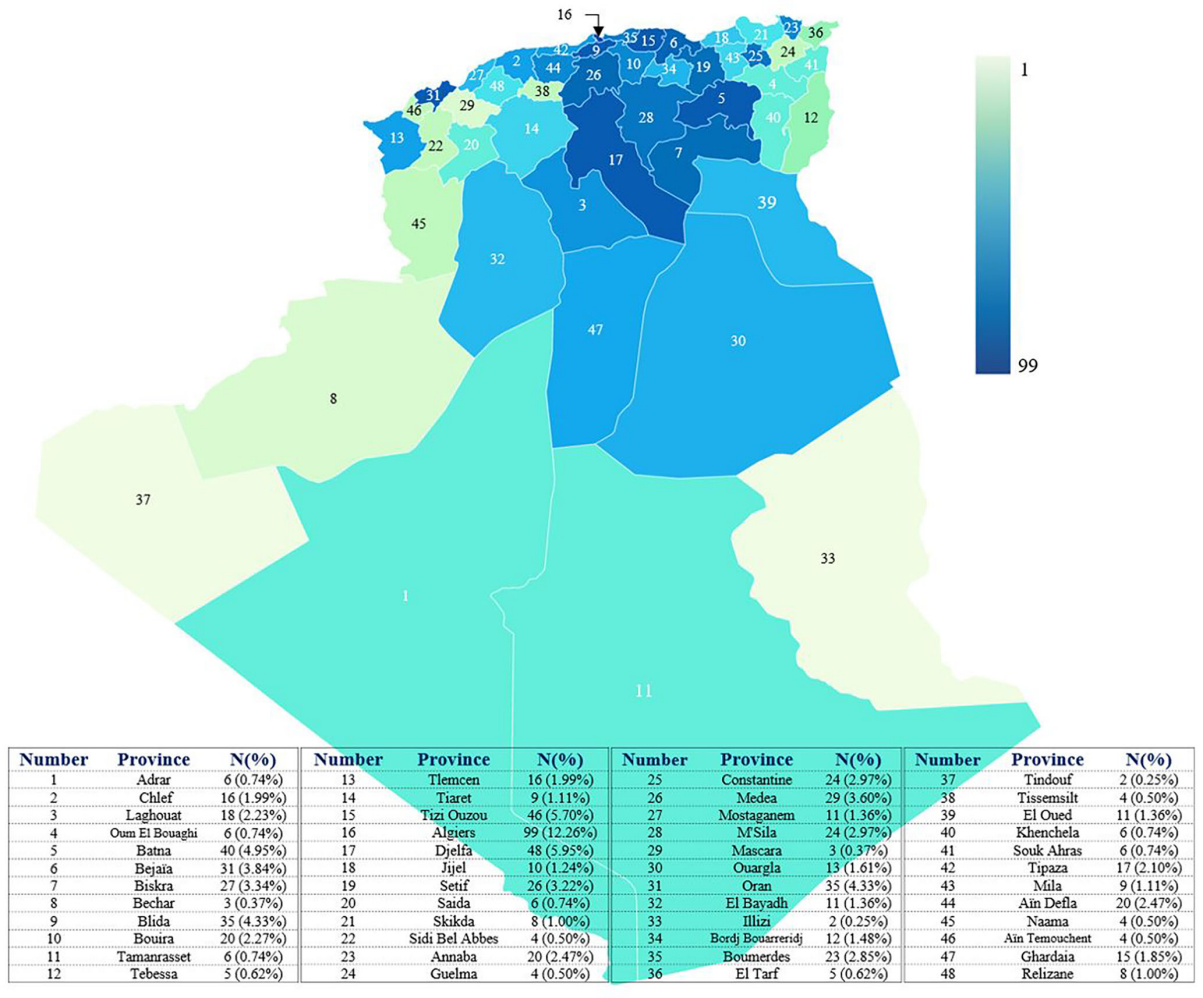
distribution of patients in the different provinces of Algeria. The base map was created with the permission of data wrapper. The intensity of the blue color is proportional to the number of patients. The registered number includes living and deceased patients. Each number on the map corresponds to a province.

The minimum overall IEI prevalence in the Algerian population was estimated at 1.8/100,000 inhabitants, with drastic variations among the provinces (from 0.38 to 4.2 per 100,000). From 2008 to 2021, an average of 57 new patients were made per year with 29.5% of the cohort being diagnosed from 2008 to 2016, and 70.5% from 2017 to September 2021. From 2019, more than 120 new cases are diagnosed every year (**Figure 4**; **Supplementary Figure S2**), the highest number was recorded in 2019. On the basis of the patient’s home address, the geographical distribution of patients was heterogeneous with the large number of cases was found in the following provinces (**Figure 5**): Algiers, Djelfa, Tizi ouzou, and Batna.

### Clinical Features

At time of diagnosis, patients with IEI disorders presented with a broad clinical spectrum (**Table 2**). Recurrent infections were the most common indication for investigation, and respiratory tract infections were the most frequent. The least common infections were osteoarticular infections, septicemia, and meningitis/encephalitis. CID was the most category affected by recurrent infections, followed by PAD and syCID. Lower respiratory tract infections predominated in CIDs, PADs, syCIDs, and phagocytic disorders, with 73.2%, 71.7%, 57.2%, and 43.9%, respectively, whereas, upper respiratory tract infections found more frequently in PADs (54%), gastrointestinal infections in CIDs (45%), and skin infections (usually deep or superficial abscesses) in phagocytic disorders (43.9%). With respect to *Mycobacterium bovis* disease, as a serious complication of Bacillus Calmette-Guérin (BCG) vaccination, ranging from local disease (known as BCGitis) to disseminated disease (BCGosis), occurred in twelve cases with CID, nine cases with inborn defects of the IL-12/IFN-γ axis, three cases with syCID, and one case with phagocytic disorder. Failure to thrive and growth retardation were observed in 82 patients (10.1%). Bronchiectasis was found to be a common complication of PADs (21.7%).

**Table 2:** Clinical presentations and complications of IEIs observed in Algerian patients.

Clinical presentation	All IEIsN=807 (%)	CIDN=273 (%)	SyCIDN=194 (%)	PADN=198 (%)	DIDN=50 (%)	PDN=57 (%)	Other IEIsN=35 (%)
**Upper respiratory tract infections**	259 (32%)	74 (27.1%)	51 (26.3%)	107 (54%)	14 (28%)	10 (17.5%)	3 (8.6%)
**Lower respiratory tract infections**	497 (61.5%)	200 (73.2%)	111 (57.2%)	142 (71.7%)	12 (24%)	25 (43.9%)	7 (20%)
**Skin infections**	119 (14.7%)	45 (16.5%)	27 (13.9%)	12 (6%)	5 (10%)	25 (43.9%)	5 (14.3%)
**Gastrointestinal tract infections**	219 (27.1%)	123 (45%)	53 (27.3%)	10 (5%)	9 (18%)	17 (29.8%)	7 (20%)
**Urinary tract infections**	49 (6%)	22 (8%)	13 (6.7%)	10 (5%)	2 (4%)	2 (3.5%)	0 (0%)
**Septicemia**	26 (3.2%)	11 (4%)	4 (4.2%)	4 (2%)	3 (6%)	3 (5.3%)	1 (2.9%)
**Candidiasis infections**	146 (18%)	95 (34.8%)	26 (13.4%)	13 (6.5%)	5 (10%)	1 (1.8%)	6 (17.1%)
**Meningitis/encephalitis**	29 (3.5%)	14 (5.1%)	2 (1%)	6 (3%)	2 (4%)	4 (7%)	1 (2.9%)
**Osteoarticular infections**	3 (0.3%)	1 (0.3%)	1 (0.5%)	1 (0.5%)	0 (0%)	0 (0%)	0 (0%)
**Failure to thrive/growth retardation**	82 (10.1%)	43 (15.7%)	12 (6.2%)	13 (6.5%)	4 (8%)	7 (12.3%)	3 (8.6%)
**BCGitis/BCGosis**	26 (3.2%)	12 (4.4%)	3 (1.5%)	0 (0%)	1 (2%)	1 (1.8%)	9 (25.7%)
**Bronchiectasis**	64 (7.9%)	14 (5.1%)	6 (3%)	43 (21.7%)	0 (0%)	1 (1.8%)	0 (0%)
**Lymphadenopathy**	98 (12.1%)	41 (15%)	15 (7.7%)	16 (8%)	14 (28%)	9 (15.8%)	3 (8.6%)
**Hepatomegaly**	64 (7.9%)	19 (7%)	8 (4.1%)	14 (7%)	17 (34%)	4 (7%)	2 (5.7%)
**Splenomegaly**	127 (15.7%)	38 (14%)	19 (9.8%)	25 (12.6%)	35 (70%)	6 (10.5%)	4 (11.4%)
**Autoimmunity**	76 (9.4%)	14 (5.1%)	18 (9.2%)	25 (12.6%)	17 (34%)	0 (0%)	2 (5.7%)

CID, combined immunodeficiencies; DID, Dysregulation of immune diseases; PAD, predominantly antibody deficiencies; PD, phagocytic disorders; SyCID, syndromic combined immunodeficiencies; other IEIs includes, complement deficiencies, innate immune deficiencies, autoinflammatory diseases, and Somatic phenocopies.

Autoimmune disorders, including autoimmune hemolytic anemia, thrombocytopenia, neutropenia, organ-specific autoimmune diseases, coeliac disease, Sjogren’s syndrome, were globally less common and were observed in 76 of total (9.4%), while they were relatively more common in immune dysregulation disorders (34%). Patients presented with splenomegaly, often accompanied by hepatomegaly and/or multifocal peripheral and/or internal lymphadenopathy, and these manifestations were found to be more frequent in patients with immune dysregulation disorders than those in other categories.

### Mutational Analysis

Genetic testing was performed in 287 patients (35.5%), among them 129 patients were confirmed carrying pathogenic germline variants (**Table 3**), of which 22 (18.5%) were located in *RFXANK*, 14 (12.6%) in *RAG1*, 13 (10.9%) in *RAG2*, 9 (7.5%) in *BTK*, 5 (4.2%) in *DOCK8*, 5 (4.2%) in *STAT3*, 5 (4.2%) in *RFXAP, 5(4.2%)* in *CFTR*, 4 (3.3%) in *RIPK1*, 4 (3.3%) in *ITGB2*, 3 (2.5%) in *STAT1 (GOF)*, 3 (2.5%) *in RAB27A*, 3 (2.5%) in *ADA*, 3 (2.5%) in *IKBKB*, 2 (1.7%) in *DCLRE1C*, 2 (1.7%) in *22q11.2del*, 2 (1.7%) in *IL12RB1*, 2 (1.7%) in *CD40*, 2 (1.7%) in *JAK3* and in other genes (*IL2RG, CD40L, CD3Z, 11q23del, CD79A, CARD11, CD19, ORAI1, IGHM, TCF3, NFKB2, TNFRSF6, UNC13D, FERMT3, MVK, CYBB, GATA2, IRF3*) 1 (0.8%) for each of these genes. However, in some cases more than one pathogenic mutation in different genes have been identified. The first case was a rare double homozygosity of two different mutations in two brothers, a missense biallelic variation in the *CD3G* gene and a biallelic nucleotide deletion mutation in the *CD3E* gene. The two mutations were considered as previously unreported and were expected to cause the disorder. The second case concerned a 16-month-old boy who also carried two variations, a heterozygous nonsense nucleotide substitution mutation in the *NOD2* gene, and a homozygous nucleotide deletion mutation in the *RFXANK* gene, responsible of Blau syndrome and MHC class II deficiency, respectively. Both mutations have been previously reported and recognized to be pathogenic. Most of the remaining patients are still under analysis.

**Table 3:** distribution of patients according to genetically-confirmed patients.

Category of IEI	Diagnosis	Inheritance	Gene identified	Number of patients
**Combined immunodeficiencies**	Severe combined immunodeficiency	AR/homozygous	*RAG1*	5
		AR/homozygous	*RAG2*	11
		AR/homozygous	*DCLRE1C*	1
		AR/homozygous	*ADA*	3
		AR/homozygous	*JAK3*	2
	Hypomorphic Severe combined immunodeficiency	AR/homozygous	*RAG1*	1
		AR/homozygous	*RAG2*	1
		AR/homozygous	*DCLRE1C*	1
		XLR	*IL2RG*	1
	Omenn syndrome	AR/homozygous	*RAG1*	8
		AR/homozygous	*RAG2*	1
	Hyper-IgM syndrome	AR/homozygous	*CD40*	2
		XLR	*CD40L*	1
	Partial CD3 chain deficiencies	AR/homozygous	*CD3Z*	1
		AR/homozygous	*CD3G/CD3E*	2
	MHC-II deficiency	AR/homozygous	*RFXANK*	22
			*RFXAP*	5
	MHC-II deficiency/Blau syndrome	AR/homozygous	*RFXANK/NOD2*	1
	DOCK8 deficiency	AR/homozygous	*DOCK8*	5
	IKBKB deficiency	AR/homozygous	*IKBKB*	3
**Syndromic combined immunodeficiencies**	Di George syndrome	AD	*11q23del*	2
	Calcium channel defect	AR/heterozygous	*ORAI-1*	1
	Jacobsen syndrome	AD	*11q23del*	1
	Hyper-IgE syndrome	AD (LOF)	*STAT3*	5
		AD (LOF)	*CARD11*	1
**Predominantly antibody deficiencies**	Congenital Agammaglobulinemia	XLR	*BTK*	9
		AR/homozygous	*CD79A*	1
		AR/homozygous	*IGHM*	1
		AR/heterozygous	*TCF3*	1
	Common variable immunodeficiency	AR/homozygous	*CD19*	1
		AD	*NFKB2*	1
**Diseases of immune dysregulation**	Autoimmune lymphoproliferative syndrome	AR/homozygous	*TNFRSF6*	1
	Griscelli syndrome	AR/homozygous	*RAB27A*	3
	Familial lymphohistiocytosis type 3	AR/homozygous	*UNC13D*	1
	Inflammatory bowel disease	AR/homozygous	*RIPK1*	4
**Phagocytic defects**	Chronic granulomatous disease	XLR	*CYBB*	1
	Leukocyte adhesion deficiency type 1	AR/homozygous	*ITGB2*	4
	Leukocyte adhesion deficiency type 3	AR/homozygous	*FERMT3*	1
	Cystic fibrosis	AR/homozygous	*CFTR*	5
	GATA2 deficiency	AD	*GATA2*	1
**Defects in intrinsic and innate immunity**	Inborn errors of IFNγ immunity	AR/homozygous	*IL12RB1*	2
	Chronic mucocutaneous candidiasis	AD (GOF)	*STAT1 (GOF)*	3
	IRF3 deficiency	AD	*IRF3*	1
**Autoinflammatory diseases**	Hyper-IgD syndrome	AR/homozygous	*MVK*	1
				Total=129

AD, autosomal dominant; AR, autosomal recessive; GOF, gain of function; LOF, loss of function; XLR, X-linked recessive.

### Mortality

**Figure 6:**
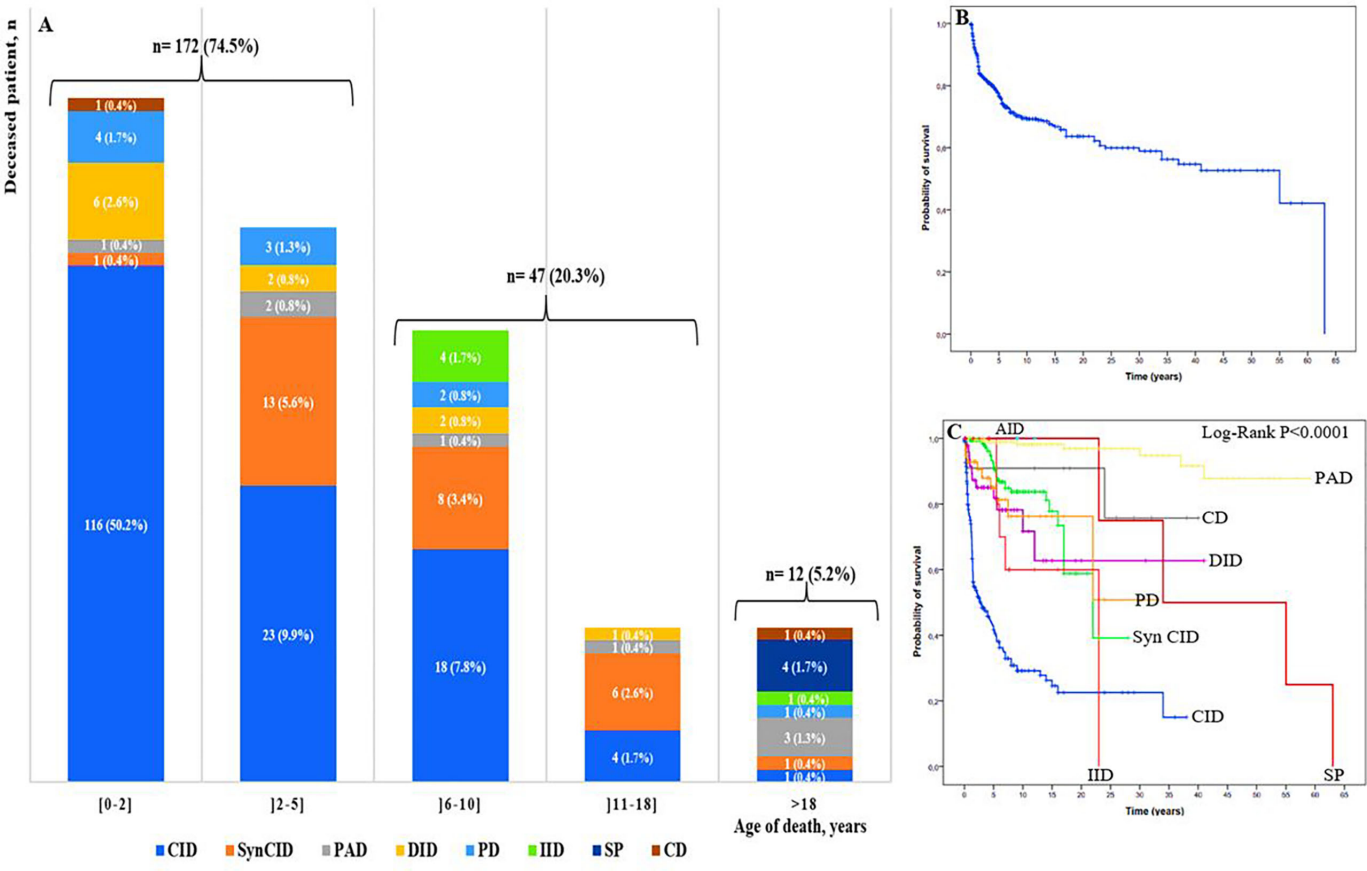
**(A)** Mortality rate by PID group and age of the deceased patients (n: 231). Colors represent different IEI categories. Numbers in the plots show the number of patients and the ratio of the deceased patients by IEI group to the total number of the deceased patients of the age-group; **(B)** Overall survival (Kaplan-Meier curve) of patients with IEI showing the probability of survival following diagnosis. **(C)** Kaplan-Meier curve showing overall survival in the 807 studied patients within the 9 categories of IEI. CID, combined immunodeficiencies; SyCID, syndromic combined immunodeficiencies; PAD, predominantly antibody deficiencies; DID, Dysregulation of immune diseases; PD, phagocytic disorders; CD, complement deficiencies; IID, innate immune deficiencies; AID, autoinflammatory diseases; SP, Somatic phenocopies.

To assess mortality, we excluded 102 patients for whom we were unable to determine their status, then we analyzed the cohort of 705 patients whose status was known, including 474 alive patients and 231 cases confirmed to be dead. Among deceased patients, 219 (94.8%) were children and 12 (5.2%) were adults (**Figure 6A**). The overall mortality rate was estimated at 32.7%. It ranged from 0.4% to 50.2% in different age ranges; the highest rate was found in children in their first 2 years of life. The majority of infant deaths occurred in patients with SCID and Omenn syndrome (92 of 129; 71.3%). In the two next age ranges (2–5 years and 6-10 years), mortality was higher in patients with CID and syndromic CID. Overall, 75% (172/231) of all IEI-related deaths occurred in patients during their first 5 years of life, whereas, in adult, the mortality rate was significantly lower, with approximately equal proportions in the following seven IEI categories: predominantly antibody deficiencies, somatic phenocopies, CID, syndromic CID, phagocytic deficiencies, complement deficiencies, and innate immune deficiencies.

The probability of survival was analyzed using the Kaplan-Meier curve, and the overall probability of survival 5 years after diagnosis was approximately 77% (**Figure 6B**), while, the lowest survival rate was 42% in CID, when comparing the survival rate in different IEI categories after the first 5 years of diagnosis, for disease of immune dysregulation was 82%, for phagocytic disorders was 84%, for syndromic CIDs and complement deficiencies was 90%, and for the predominantly antibody disorders group was 98%. The difference was found to be statistically significant between at least two categories (P<0.0001) (**Figure 6C** **;** **Supplementary Table S2**).

## Discussion

Most IEIs result from inherited defects in development and/or function of immune system; resulting in infections that develop and recur more frequently, autoimmune disorders and an increased risk of malignancies (25). However, acquired forms have also been reported (5, 26).

This current report represents a single center study which provides epidemiologic, clinical, and genetic data on Algerian patients with IEI. At the time of analysis, a total of 807 IEI patients were registered, representing all provinces of the country, thus making this cohort a valid evaluation of the IEI in Algeria. Although our department represents the largest center for immunological investigation of IEIs in Algeria, the number reported in the current study does not represent all cases, therefore we are only able to report the minimal prevalence of these conditions. It is widely accepted that IEIs are underdiagnosed worldwide, particularly in developing countries. The Algeria’s population census (up to 2021) has reported a population 45,002,737 million (21), and the overall IEI prevalence of 1.8 per 100,000 is quite low which shows that our findings certainly underestimated the disease burden in Algeria. Although our department receives the majority of patients referred from all provinces for investigation, the prevalence of these disorders is expected to be much higher in our country. This is due, on one hand, to the fact that some patients with mild forms of IEI are usually managed as outpatients without having recourse to investigation for a probable IEI but, on the other hand, severe forms of IEI such SCID and its variants usually die from severe infections before being formally diagnosed as immunodeficient patients. Also, the existence of a few other centers that participate to the investigation of IEI, all located in Algiers. Consequently, a better recruitment and diagnostic strategy could improve the coverage of diagnosed patients for a better understanding of IEI epidemiology in Algeria.

According to different IEI categories, the distribution of different types of IEI in our cohort is consistent with other reports from MENA countries. Combined immunodeficiencies (≈34%) were the most common entity of IEI, similar to the series from Tunisia, Saudi Arabia, Kuwait and Egypt (27–30), and to the MENA countries’ registry (12). Such high frequency of these disorders in our cohort could be due to ethnic/geographical particularities, and mostly to the local increased prevalence of consanguinity, and these hypotheses are strengthened by close frequencies in neighboring countries like Tunisia (27). Predominantly antibody deficiency and well-defined syndromes with immunodeficiency involved 24.5% and 24% of patients respectively, very close to IEI reports of other North African countries including Morocco, Tunisia, Egypt; except in the slight relative predominance of syndromic combined immunodeficiencies in these countries (27, 30, 31). Congenital defects of phagocytosis and immune dysregulation diseases were the fourth and fifth most common groups of IEI involving 7.1% and 6.2% of patients, respectively.

Regarding phagocytic defects, this frequency is lower than those reported from Asian countries and regions, including Qatar, Oman, China, Korea, Japan, and Asia-pacific region (16, 32–36) as well as those from Europe and Latin America but higher than those from Australia (15, 17, 37), Immune dysregulation diseases frequency is in the same range of previous studies in Saudi Arabia, Russia and Asia pacific region (16, 28, 38), while other studies showed a lower rate (15, 27, 31–33, 39–42). Severe phenotypes of IEI are more represented in our study: SCID, atypical SCID, Omenn syndrome and MHC class II deficiency were more frequent, comprising nearly ≈80% of the total combined immunodeficiencies. This might be explained by a genetic predisposition/background in the Algerian population.

The overall median diagnostic delay in all IEIs (time between symptom onset and diagnosis) was 16 months, close to that of neighboring countries: Morocco (24 months) (31), Tunisia (18 months) (27). However, patients with CID were more promptly diagnosed (mean delay =7.5 months). This is probably due to the early-onset and severe symptoms these patients do present in the first year of life, with rapid referral and a subsequent earlier age at diagnosis.

Similar to various reports (27, 29, 31, 38, 43–47), age at the onset of symptoms and at diagnosis as well as diagnosis delay were very variable according to the IEI groups. Early diagnosis and management of IEI can lead to lower morbidity and mortality rates and better quality of life in IEI patients (48).

In accordance with previous studies (49), the vast majority of IEI patients in our cohort were children (90.3%) and about 65% of patients were diagnosed before the age of 5. In some studies, adult patients (aged 18 or above) represent almost half of all IEI (32, 47). Hence, adults with IEI are the most underdiagnosed group in Algeria. This is supported by the low proportion of predominantly antibody deficiencies in our cohort compared to other countries (13, 50, 51). Of note, most of patients referred to our department came from the pediatric departments, suggesting that education sessions and programs may be required to raise awareness of IEI especially CVID in general practitioners and adult specialists.

An increased frequency of unions between relatives is associated with an increased birth prevalence of children with severe autosomal recessive single-gene disorders (52). Parental consanguinity rate in our cohort was 50.3%; higher rates have also been reported in other MENA countries as in Kuwait (29), Oman (33), Saudi Arabia (28), Egypt (30), Qatar (36), Iran (41), Tunisia (27) and Algeria (53) at a rate of 78, 76, 75, 75.2, 61.1, 60.1, 58.2%, and 52.6%, respectively. But it is significantly lower than that described in other countries such as Russia (38), Germany (47), US (14), UK (46), France (43), South Africa (44), estimated at 1.6, 8, 10, 15, and 1.2%, respectively. There was also a notable difference in consanguinity rates between the nine groups among which the highest level was found in immune dysregulation diseases with 74%, while its frequency was lower in complement deficiencies with 18.1%. Although X-linked conditions such as XLA and X-linked SCID are less common in our cohort, the male ratio in different category of IEI does not differ greatly from previous studies, with males predominating amongst children and adult patients which is in line with worldwide trends (12, 14, 15, 17, 32, 34, 37–39, 41, 44, 46, 47).

The increased diagnosis over the last decade reflects a major and active effort to improve the field of clinical immunology by organizing a working group studying IEIs in our country, the organization of scientific events and campaigns about IEI for a better awareness and understanding of IEI presentations and the existence of a specialized laboratory in the investigation of IEIs at Beni Messous university hospital center in Algiers.

Recurrent and chronic infections, rather than atypical infections, are the main clinical feature of IEI in this cohort. Several cohort studies, including large IEI patients, indicated that the majority of patients had a history of repeated and/or persistent infections before a definitive diagnosis was made (54–56). In consistent with the reported studies, the present cohort revealed that more than 90% of IEI patients presented symptoms of recurrent infections before diagnosis. The most common infections are respiratory tract infections (lower 61.5% and upper 32%), followed by gastrointestinal tract infections (27.1%). It is not surprising that most of infectious agents invade the human body through the respiratory and digestive tract. Indeed, the mucosal surfaces of the body are particularly vulnerable to infection especially in patients with altered immunity such as people with IEI, cancer or HIV/AIDS, because mucosal surfaces especially bronchus-associated lymphoid tissue (BALT) or gastric-associated lymphoid tissue (GALT) are constantly exposed to pathogenic or nonpathogenic micro-organisms (57). This suggests that in such clinical manifestations, immunological assessment is required mainly when they occur early in life. Moreover, gastrointestinal diseases related to IEI are not only caused by infection, but also by inflammatory or autoimmune process, or malignancy. Sometimes, recurrent and/or persistent digestive symptom could be the unique presentation of IEI, thus it will be necessary to pay more attention of the possibility of IEI in patient with chronic diarrhea, growth retardation and failure to thrive especially the ones who do not respond to conventional therapy (58, 59). Most of localized and disseminated BCG infections have occurred in patients with combined immunodeficiency and with inborn defects of the IFNγ/IL12 axis which found to be in agreement with previously reported findings (60–62). As BCG vaccine is given to infants at birth in Algeria, serious adverse reactions after BCG vaccination may serve as a useful clinical signature to consider investigation of IEI in such children (63). BCG vaccination should be avoided if any family history of IEI or early deaths from infections or complication of tuberculosis. In addition to unusual increased susceptibility to infections, various noninfectious disorders can also occur in IEI patients such as autoimmune disorders and malignancies among others (64, 65). In this cohort, autoimmune diseases and lymphoproliferative disorders appeared to be more frequent in diseases of immune dysregulation. Studies of apoptosis and tolerance of T cells especially in patients with monogenic defects allowed us to understand self-tolerance, occurrence of lymphoproliferative syndrome, and autoimmune phenomena (66).

There were no facilities that offer regular genetic testing in Algeria, until recently, where our department was equipped with a platform of molecular genetics and cytogenetics, but these are not yet fully operational. Besides some research projects carried out locally, most molecular diagnostics were performed abroad, through international collaborations. Therefore, only 16% of all patients included in this study had a genetic diagnosis. This percentage is most similar to the Tunisian report (27), but lower compared to other reports (29, 36, 38, 41, 43). This could be due to the fact that the diagnosis is easily made based on clinical and laboratory findings, and identification of the genetic defect will not have any effect on the further treatment. On other hand, genetic testing was not carried out in some IEI such as common variable immunodeficiencies, selective IgA deficiency, and some other unclassified IEIs. This is partly due to the fact that genetic defects often go undetected, even using next generation sequencing methods (67). Unsurprisingly, the majority of the genetic disorders described have autosomal recessive inheritance and occur in the homozygous state for most of the respective mutations. This could be explained by the founder effect, known to underlie several IEI genes in highly consanguineous populations such as ours (68).

**Figure 7:**
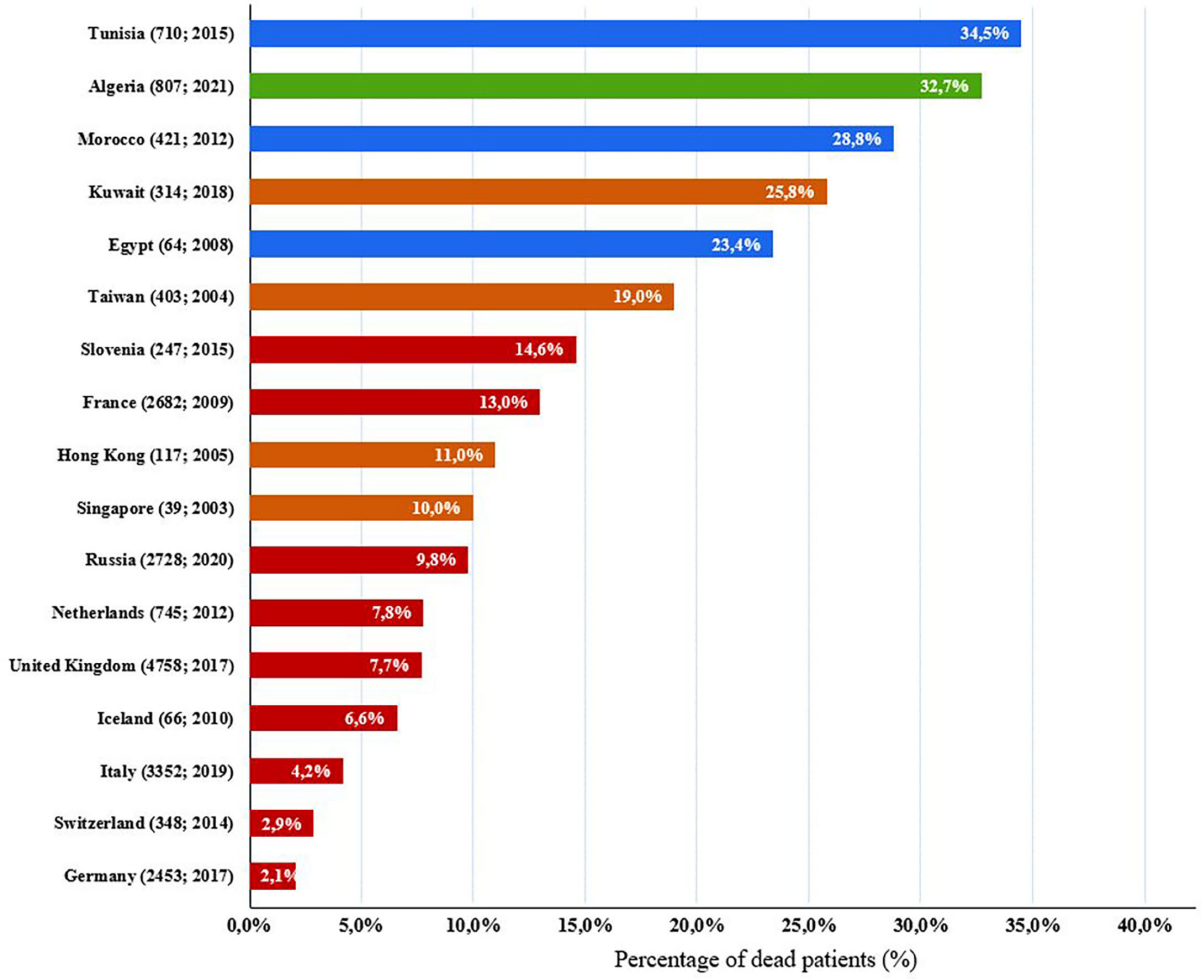
Comparison of IEI death percentages in our cohort with previously published IEI studies of other countries. Algeria: green; European countries: red; Asian countries and regions: brown, north African countries: blue. Parentheses next to each country name correspond to number of patients followed by year of analysis (number of patients; year of analysis).

In this study, we reported mortality rate of 32.7%, with severe infections as the most leading causes of death. Overall, the mortality of our cohort is comparable to the findings of neighboring countries such as Morocco (31) and Tunisia (27), but it is much higher than to what was reported in European countries (38, 43, 45–47, 69–72) and Asian countries and regions (73–75) (**Figure 7**). Furthermore, Combined immunodeficiency is the category with the highest mortality rate (70.1%) which is very close to what was found in Kuwait 69% (29). This is due to the high incidence rate of SCID associated with a high frequency and severity of infections. Hence, these patients ultimately die generally during their first year of life, due to the lack of allogeneic stem cell transplantation or gene therapy in our country. The variability in mortality rates, reported in different reports, depends on the type of IEI most common in the patient’s country or region, and also on resource availability for early diagnosis (newborn screening), as well as the supply of proper treatment such as hematopoietic stem cell transplantation (HSCT) which is the mainstay of curative treatment in CID patients. There is little information on survival of patients with IEI. The management of these patients is challenging in developing countries, and this has resulted in high mortality rate. This study showed an overall 5-year survival rate of 77%, ranging from the highest in antibody deficiencies, at 98% and lowest in combined immunodeficiencies, at 42%. Similar findings have been shown in Oman (33) and Iran (41). Such findings are useful when describing resources for IEI patients, awareness raising, and its management.

## Conclusion

Our study represents a sample of children and adult Algerian patients carrying a wide spectrum of IEI.

This monocentric cohort showed that IEI mostly appear at an early age, and recurrent/severe infections were the most common clinical manifestations in Algeria, and late diagnosis (or even misdiagnosis) can be disastrous and lead to higher morbidity and mortality.

The study confirmed, in a highly consanguineous population, the increased frequency of the autosomal recessive IEI, along with peculiar clinical features and genetic characteristics.

Increasing awareness and education of medical practitioners, expanding diagnostic tests and establishing a national hematopoietic stem cell transplantation (HSCT), are urgently needed to reduce the heavy burden of IEI in Algeria.

## Data Availability Statement

The original contributions presented in the study are included in the article/**Supplementary Material**. Further inquiries can be directed to the corresponding author.

## Ethics Statement

The studies involving human participants were reviewed and approved by The ethics committee of the Beni Messous university hospital center. Written informed consent to participate in this study was provided by the participants’ legal guardian/next of kin.

## Author Contribution

BBe, RD conceptualized the study. BBe, LL-M, FM, LB, ZL, IA performed data analysis under supervision by RD. LB, K-WC, DL, AP, JC and YL performed genetic study. BBe drafted the manuscript. Other authors referred patients and provided clinical care and clinical data. All authors critically reviewed the manuscript and approved the submitted version.

## Conflict of Interest

The authors declare that the research was conducted in the absence of any commercial or financial relationships that could be construed as a potential conflict of interest.

## Publisher’s Note

All claims expressed in this article are solely those of the authors and do not necessarily represent those of their affiliated organizations, or those of the publisher, the editors and the reviewers. Any product that may be evaluated in this article, or claim that may be made by its manufacturer, is not guaranteed or endorsed by the publisher.

## Abbreviations

ALPS, autoimmune lymphoproliferative syndrome; AD, Autosomal dominant; AR, Autosomal recessive; CD, cluster of differentiation; CHARGE, coloboma, choanal atresia, heart defects, genital abnormalities, retarded growth, and ear malformation; Chr, Chromosome; CID, combined immunodeficiency; CGD, chronic granulomatous disease; CVID, common variable immunodeficiency; BAFF-R, B cell-activating factor receptor; DHR, Dihydrorhodamin; DOCK8, Dedicator of cytokinesis 8; GOF, Gain of function; HIGMS, Hyper-IgM syndrome; HLA, Human leukocyte Antigen; HLH, hemophagocytic lymphohistiocytosis; IKBKB, Inhibitor Of Nuclear Factor Kappa B Kinase Subunit Beta; IUIS, International union of immunological societies; LAD, leukocyte Adhesion deficiency; LOF, loss of function; MENA, Middle East and North Africa.; MHC, major histocompatibility complex; MSMD, mendelian susceptibility to mycobacterial disease; NFKB, nuclear factor kappa-light-chain-enhancer of activated B cells; PID, primary immune deficiency; SCID, Severe combined immunodeficiency; XLA, X-linked agammaglobulinemia; XLT, X linked thrombocytopenia; WAS, Wiskott-Aldrich Syndrome; ZAP70, Zeta associated protein 70.
