# Supplementary figures and images for: Inborn Errors of Immunity in Algerian Children and Adults: A Single-Center Experience Over a Period of 13 Years (2008–2021)
Source: Front Immunol. 2022 Apr 21;13:900091. doi: 10.3389/fimmu.2022.900091 (PMC9069527; doi:10.3389/fimmu.2022.900091)

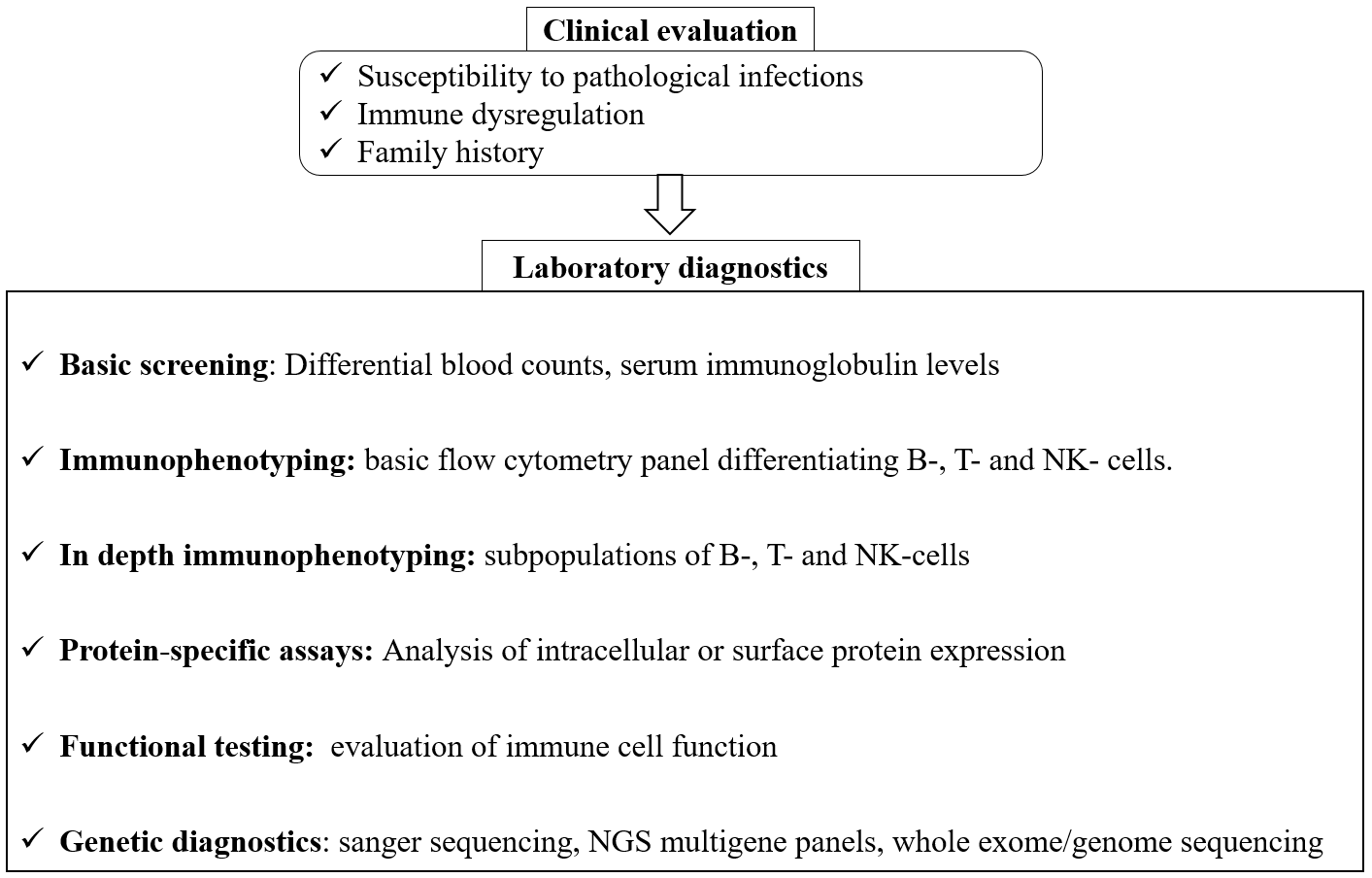


**Supplemtary figure S1**: Flow chart showing immunological diagnosis of IEIs

Supplement: Supplementary file 1 [file DataSheet_1.docx]

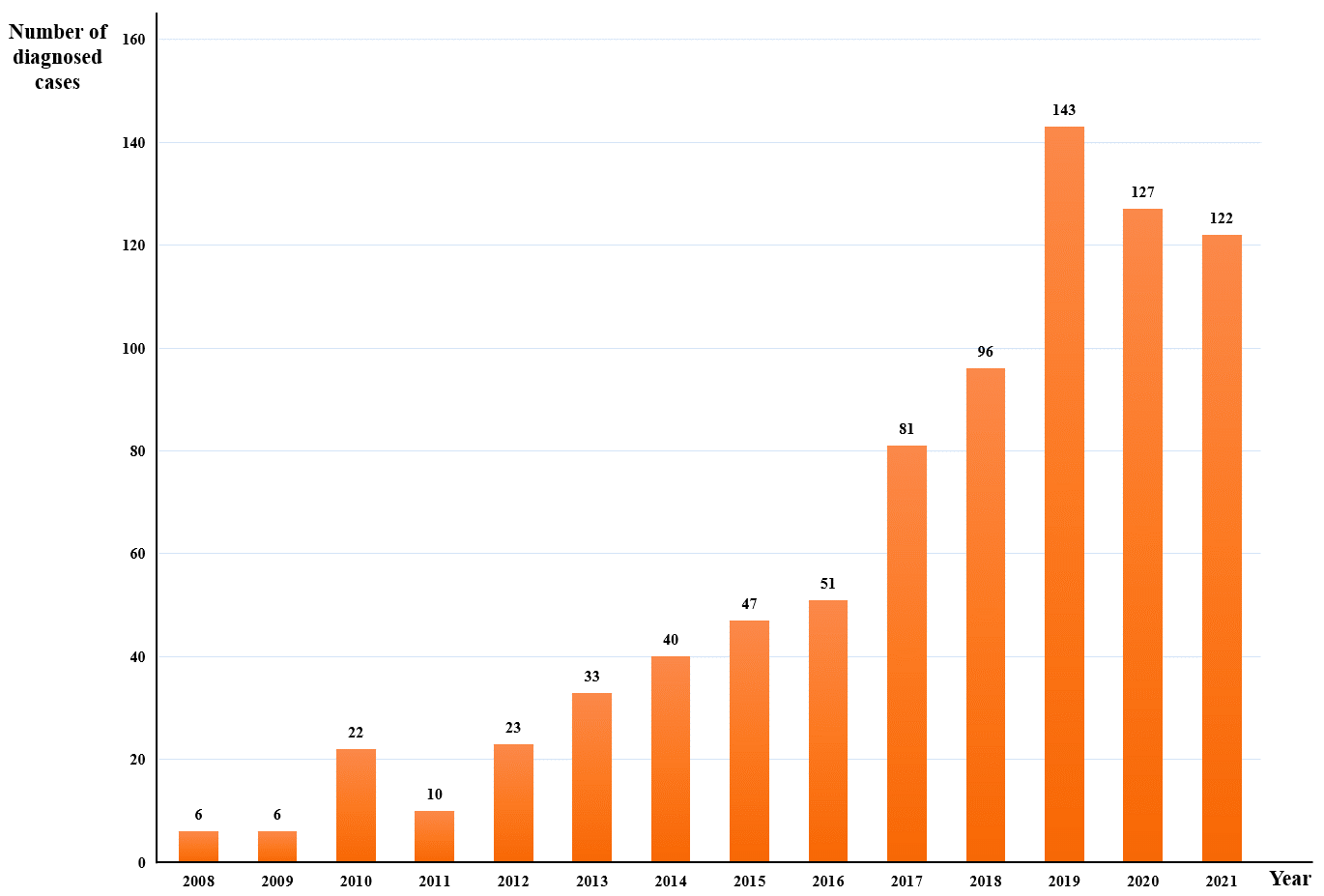


**Supplementary Figure S2**: Number of new PID diagnoses per year from 2008 to September 2021

Supplement: Supplementary file 2 [file DataSheet_2.docx]
