# Supplementary material for: Inborn Errors of Immunity in Algerian Children and Adults: A Single-Center Experience Over a Period of 13 Years (2008–2021)
Source: Front Immunol. 2022 Apr 21;13:900091. doi: 10.3389/fimmu.2022.900091 (PMC9069527; doi:10.3389/fimmu.2022.900091)
Supplement: Supplementary file 3 [file Table_1.docx]

**Supplementary table S1:** Flow cytometry panels for primary immunodeficiency disease diagnosis

| Panels/Test | Target protein (clone)/product |
| --- | --- |
| T, B and NK cell panel | CD3 (SK7), CD19 (SJ25C1), CD56 (NCAM16.2), CD16(B73.1), CD4 (SK3), CD8 (SK1) |
| Extended T cell panel | CD45RA (HI100), CD45RO (UCHL1), CCR7 (150503) |
| Extended B cell panel | CD19 (SJ25C1), CD24 (SN3), CD38 (T16), CD27 (0323), IgD (IA6-2) |
| Recent thymic Emigrant (RTE) panel | CD3 (SK7), CD4 (SK3), CD45RA (HI100), CD31 (L133.1) |
| HLA-DR panel | CD19(SJ25C1), HLA-DR(L243) |
| HLA-ABC panel | CD3(SK7), HLA-ABC(DX17) |
| Lymphocyte proliferation assay panel | CFSE, 7AAD, CD3 (SK7) |
| Double negative T-cell panel, ALPS | CD3(SK7), CD4(SK3), CD8(SK1), TCR-αβ (WT31), TCR-γδ (011F2) |
| BTK panel, XLA | BTK (53), CD14 (MφP9) |
| Bone morrow precursor B cells, ARA | CD79a(HM47), CD79b(3A2-2E7), IgM(G20-127), BLNK(B11), CD179a(HSL96) |
| WASp panel, WAS | WASP (5A5), CD3(SK7) |
| pSTAT1 panel, MSMD/STAT1GOF | CD3(UCHT1), CD4(SK3), pSTAT-1 pY701 (4a) |
| IFNγRα, MSMD | CD14(MφP9), CD119(GIR-94) |
| IL12Rβ1, MSMD | CD3 (SK7), CD4 (SK3), CD25 (2A3), CD212 (2.4E6) |
| pSTAT3 panel | CD3(UCHT1), CD4(SK3), pSTAT-1 pY705 (4/P-STAT3) |
| IL6ST, IL6RA, HIES | CD3(SK7), CD4(SK3), IL6st (AM64), IL6ra(M5) |
| pSTAT5 panel | CD3(UCHT1), CD4(SK3), CD8(RPA-T8), pSTAT-1 pY694 (47) |
| Gamma common chain, SCID panel | CD19(SJ25C1), CD132(AG184) |
| Perforin, HLH panel | CD56 (NCAM16.2), Perforin (8G9) |
| SAP, XIAP, XLP 1/2 panel | CD3(SK7), CD8(SK1), CD56(NCAM16.2), SAP(1A9), XIAP(28/hILP/XIAP) |
| CD18, CD15, LAD panel | CD18(L130), CD15(MMA) |
| Degranulation assay | CD56(NCAM16.2), CD107a(H4A3), K562 target cells |
| Cytotoxicity assay | NKTEST^TM^ |
| Neutrophil respiratory burst test, CGD | DHR 123, NADPH oxidase |
| P47phox, P67phox, CGD panel | CD15(MMA), P47phox (1/p47Phox), P67phox(9/p67phox) |
| Hyper-IgM panel | CD40 (5C3), CD40L (TRAP1) |
| CD46, aHUS | CD3(SK7), CD14(MφP9), CD15(MMA), CD46(E4.3) |
| Regulatory T cell panel | FOXP3 (PCH101), CD25 (2A3), CD4 (RPA-T4), CD127(HIL-7R-M21) |
