# Supplementary material for: Inborn Errors of Immunity in Algerian Children and Adults: A Single-Center Experience Over a Period of 13 Years (2008–2021)
Source: Front Immunol. 2022 Apr 21;13:900091. doi: 10.3389/fimmu.2022.900091 (PMC9069527; doi:10.3389/fimmu.2022.900091)
Supplement: Supplementary file 4 [file Table_2.docx]

| Group | | **combined immunodeficiencies (CIDs)** | | **Syndromic immunodeficiencies**  **(SyCIDs)** | | **predominantly antibody deficiencies**  **(PADs)** | | **Disease of immune dysregulation**  **(DIDs)** | | **phagocytic disorders**  **(PD)** | | **innate immune deficiencies**  **(IID)** | | **autoinflammatory diseases**  **(AID)** | | **complement deficiencies**  **(CD)** | | **Somatic phenocopies**  **(SP)** | |
| --- | --- | --- | --- | --- | --- | --- | --- | --- | --- | --- | --- | --- | --- | --- | --- | --- | --- | --- | --- |
|  |  | Khi-deux | p | Khi-deux | p | Khi-deux | p | Khi-deux | p | Khi-deux | p | Khi-deux | p | Khi-deux | p | Khi-deux | p | Khi-deux | Sig. |
| Log Rank (Mantel-Cox) | CIDs |  |  | 121,569 | ,000 | 210,127 | ,000 | 23,052 | ,000 | 26,274 | ,000 | 6,969 | ,008 | 4,939 | ,026 | 11,019 | ,001 | 4,096 | ,043 |
|  | SyCIDs | 121,569 | ,000 |  |  | 39,236 | ,000 | 2,341 | ,126 | ,878 | ,349 | 1,249 | ,264 | ,708 | ,400 | 2,861 | ,091 | 2,035 | ,154 |
|  | PADs | 210,127 | ,000 | 39,236 | ,000 |  |  | 38,661 | ,000 | 35,287 | ,000 | 52,920 | ,000 | ,073 | ,786 | 4,394 | ,036 | 6,788 | ,009 |
|  | DID | 23,052 | ,000 | 2,341 | ,126 | 38,661 | ,000 |  |  | ,107 | ,744 | ,135 | ,713 | 1,176 | ,278 | 1,364 | ,243 | ,472 | ,492 |
|  | PD | 26,274 | ,000 | ,878 | ,349 | 35,287 | ,000 | ,107 | ,744 |  |  | ,375 | ,540 | 1,063 | ,303 | 1,209 | ,272 | 1,066 | ,302 |
|  | IID | 6,969 | ,008 | 1,249 | ,264 | 52,920 | ,000 | ,135 | ,713 | ,375 | ,540 |  |  | 1,937 | ,164 | 4,037 | ,045 | 3,261 | ,071 |
|  | AID | 4,939 | ,026 | ,708 | ,400 | ,073 | ,786 | 1,176 | ,278 | 1,063 | ,303 | 1,937 | ,164 |  |  | ,364 | ,546 |  |  |
|  | CD | 11,019 | ,001 | 2,861 | ,091 | 4,394 | ,036 | 1,364 | ,243 | 1,209 | ,272 | 4,037 | ,045 | ,364 | ,546 |  |  | ,178 | ,673 |
|  | SP | 4,096 | ,043 | 2,035 | ,154 | 6,788 | ,009 | ,472 | ,492 | 1,066 | ,302 | 3,261 | ,071 |  |  | ,178 | ,673 |  |  |

**Supplementary table S2:** Comparison of mortality rates between different group of patients with IEI.
